# Gas-Phase Production of Hydroxylated Silicon Oxide Cluster Cations: Structure, Infrared Spectroscopy, and Astronomical Relevance

**DOI:** 10.1021/acsearthspacechem.3c00346

**Published:** 2024-05-09

**Authors:** Andreu A. de Donato, Bianca-Andreea Ghejan, Joost M. Bakker, Thorsten M. Bernhardt, Stefan T. Bromley, Sandra M. Lang

**Affiliations:** † Departament de Ciència de Materials i Química Física & Institut de Química Teòrica i Computacio-nal (IQTCUB), Universitat de Barcelona, c/Martí i Franquès 1-11, Barcelona 08028, Spain; ‡ Institute of Surface Chemistry and Catalysis, University of Ulm, Ulm 89069, Germany; § Radboud University, Institute of Molecules and Materials, FELIX Laboratory, Nijmegen 6525 ED, The Netherlands; ∥ Institució Catalana de Recerca i Estudis Avançats (ICREA), Passeig Lluís Companys 23, Bar-celona E-08010, Spain

**Keywords:** silicon oxide clusters, hydroxylation, infrared photodissocation spectroscopy, interstellar dust formation, astrochemistry, density functional theory

## Abstract

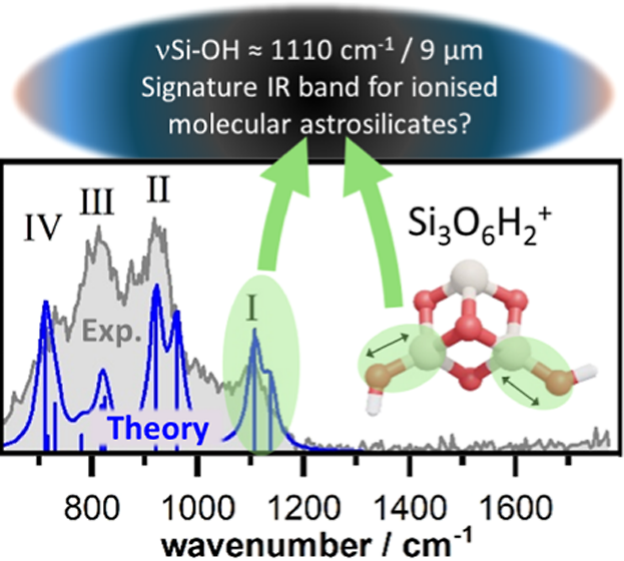

The interaction of free cationic silicon oxide clusters, Si_*x*_O_*y*_^+^ (*x* = 2–5, *y* ≥ *x*), with dilute water vapor, was investigated in a flow tube reactor. Product mass distributions indicate cluster size-dependent dissociative water adsorption. To probe the structure and vibrational spectra of the resulting Si_*x*_O_*y*_H_2_^+^ (*x* = 2–4) clusters, we employed infrared multiple photon dissociation spectroscopy and density functional theory calculations. The planar rhombic cluster core of the disilicon oxides (*x* = 2) appears to be retained upon dissociative adsorption of one H_2_O unit, whereas a significant structural transformation of the tri- and tetra-silicon oxides (*x* = 3 and 4) is induced, resulting in an increased coordination of the Si atoms and more 3D cluster structures. In an astronomical context, we discuss the potential relevance of Si_*x*_O_*y*_H_*z*_^+^ clusters as seeds for dust nucleation and catalysts for carbon-based chemistry in diffuse or translucent interstellar clouds, where all the necessary conditions for producing these species are found. In the produced clusters, the frequency of the isolated silanol Si–OH stretching vibrational mode is considerably blue-shifted compared to that in hydroxylated bulk silica and small inorganic compounds. This mode has a characteristic frequency range between 1200 cm^–1^ (8.3 μm) and 1090 cm^–1^ (9.2 μm) and is associated with the anomalously small Si–OH bond lengths in these ionised species. In infrared observations such high frequency Si–O stretching modes are usually associated with a pure bulk silica component of silicate cosmic dust. The presence of Si_*x*_O_*y*_H_2_^+^ clusters in low silica astrophysical environments could thus potentially be detected via their signature Si−O band using the James Webb space telescope.

## Introduction

Interactions between water and silicates play an essential role in many environmentally and technologically important processes.^1^ During dissolution and/or nucleation processes in aqueous solutions, small, hydroxylated silicon oxide oligomers are produced. These species tend to be slightly negatively charged due to the weak tendency for deprotonation via the dissociation of pendant hydroxyl (Si–OH) groups in aqueous solutions. As such, most experimental studies of Si_*x*_O_*y*_H_*z*_ oligomers have tended to focus on solvated anionic species either in situ via ^29^Si NMR spectroscopy^2^ or *ex situ* via mass spectrometric (MS) characterization of anionic species extracted from solution.^3^ From such studies, it has been found that Si_*x*_O_*y*_H_*z*_^–^ oligomers display a rapidly increasing structural complexity with size, ranging from simple linear species to cage-like clusters. The structures of these species and how they form and interact seems to be intimately linked to the presence of an aqueous environment (e.g., fluxionality^4,5^) and its properties (e.g., the presence of dissolved cations^6^). Controlling and understanding these factors is key for their use as building blocks for the synthesis of nanoporous silicate materials such as zeolites.^7^

The chemistry of Si_*x*_O_*y*_H_*z*_ species is also likely to be relevant to diffuse astrophysical environments. Here, the near vacuum conditions and extreme temperatures are likely to favor alternative species (e.g., charge, stoichiometries, and structures) to those found in terrestrial environments. For example, in the diffuse interstellar medium (ISM), there is a constant flux of high energy ultraviolet (UV) radiation which tends to lead to smaller species being positively ionized.^8^ UV irradiation is also known to reduce the number of OH groups on silica surfaces.^9^ Thus, in contrast to hydrated silica oligomers on earth, any small Si_*x*_O_*y*_H_*z*_ species in the diffuse ISM are more likely to be cationic and are relatively weakly hydroxylated.

In the laboratory, small gas phase Si_*x*_O_*y*_H_*z*_ clusters can be generated by laser ablation or sputtering of solid Si-based targets, avoiding the influence of aqueous solvation. A range of Si_*x*_O_*y*_H_*z*_ compositions for both cationic^10−13^ and anionic^14−16^ clusters has been produced in MS experiments, which can provide elemental compositions. On its own, however, MS provides no structural information, and the structures of the produced clusters can only be tentatively assigned based on theoretical calculations.^10−12,15−17^ Infrared (IR) spectroscopy can provide a vibrational fingerprint of gas phase-produced clusters. Accurately calculated IR spectra of low energy cluster isomers can be obtained from computational chemical modeling, which can then be used to identify the experimental cluster structures. Such collaborative studies have structurally characterized cationic bare silicon oxide species (i.e., Si_*x*_O_*y*_^+^)^18,19^ but, as yet, have not been used for hydroxylated Si_*x*_O_*y*_H_*z*_^+^ clusters.

Here, we report on the production of positively charged Si_*x*_O_*y*_H_*z*_^+^ clusters with a low degree of hydroxylation (*z* < *y*) and their structural identification via IR photodissociation spectroscopy and density functional theory (DFT) calculations. In particular, we follow the size-dependent structural and spectroscopic properties of a series of clusters with increasing silicon and oxygen content: Si_2_O_3_H_2_^+^, Si_2_O_5_H_2_^+^, Si_3_O_6_H_2_^+^, Si_4_O_7_H_2_^+^, and Si_4_O_9_H_2_^+^. In addition, we compare our findings with studies on structures of neutral Si_*x*_O_*y*_H_*z*_ and non-hydrogenated Si_*x*_O_*y*_^+^ oligomers to highlight the specific characteristics of our obtained isomers. Considering the potential astronomical relevance of these species, we discuss their possible formation routes in the ISM and characteristic IR features that could potentially be used to identify them in IR observations by the James Webb Space Telescope (JWST).

## Methodology

### Experimental Methods

Cationic silicon oxide clusters Si_*x*_O_*y*_^+^ were produced by pulsed laser ablation of a rotating silicon rod using the second harmonic of a Nd:YAG laser. The ablation took place in a 3 mm diameter, 60 mm long growth channel in the presence of a short pulse of helium carrier gas seeded with 0.15% oxygen. To induce cluster–water reactions, a mixture of 1% H_2_O in helium was introduced via a second-pulsed valve 50 mm downstream in a flow tube reactor, which was held at room temperature throughout the experiments. The reaction mixture then expanded into vacuum forming a molecular beam and interacted with the IR laser beam of the free electron laser for intra cavity experiments, (FELICE, 630–1800 cm^–1^, 10 μs macropulse with picosecond duration micropulses at 1 GHz repetition rate; spectral width set to ∼0.5% fwhm of the central frequency), crossing it at an angle of 35°. A few microseconds after interaction with FELICE, all ions were extracted by a set of pulsed high-voltage plate electrodes into a reflectron time-of-flight mass spectrometer and detected with a microchannel plate detector.^20,21^

To correct for long-term source fluctuations, the experiment was operated at twice the FELICE repetition rate, allowing for the recording of reference mass spectra between successive FELICE macropulses. Whenever the IR frequency was in resonance with an IR-active vibrational mode of a given complex, multiple IR photons were absorbed sequentially, leading to heating of the complex and finally to its fragmentation, i.e., depletion of the detected signal in the mass channel of the complex. By recording the depletion as a function of wavenumber, IR multiple-photon dissociation (IR-MPD) spectra were obtained. These are presented as the depletion yield *Y*(ν̃) at wavenumber ν̃, obtained via the equation *Y*(ν̃) = −ln[*I*(ν̃)/*I*_0_ ]/*P*(ν̃), where *I*(ν̃) and *I*_0_ are the mass channel intensities with and without laser light, respectively, and *P*(ν̃) is the macropulse energy of the FELICE IR laser.

### Computational Modeling

We obtained candidate low energy isomer structures for the five selected stoichiometries found in the experiment (i.e., Si_2_O_3_H_2_^+^, Si_2_O_5_H_2_^+^, Si_3_O_6_H_2_^+^, Si_4_O_7_H_2_^+^, and Si_4_O_9_H_2_^+^) using two global optimization search approaches using: (1) the Monte Carlo basin hopping (MCBH) algorithm^22^ and classical interatomic potentials (IPs) and (2) the first-principles energy expression (GOFEE)^23^ method.

The MCBH algorithm was implemented in the atomic simulation environment (ASE)^24^ which employed the general utility lattice program (GULP)^25^ code to perform local energy minimizations. The potential energy landscape of isomer configurations was modeled using an empirical interatomic potential based on the Buckingham form with electrostatics.^26,27^ This approach has been proven to be effective for finding low energy isomer structures for (SiO_2_)_*n*_(H_2_O)_*m*_ clusters with a range of sizes and degrees of hydration.^5,28,29^

The GOFEE approach relies on using diverse energy sampling using single-point calculations, typically performed at a DFT level of theory, to dynamically train a surrogate machine learning energy model. GOFEE can then evaluate forces and perform energy minimizations using this model with an accuracy approaching the level of the single-point sampling thus bypassing the requirement for more computationally demanding local DFT relaxations. This approach is employed to navigate the conformational space and locally relax candidate isomer structures. Various GOFEE searches were performed with energy evaluations using DFT single point calculations either with the B3LYP^30^ or PBE0^31^ hybrid exchange correlation functionals and an ultralight-tier 1 numerical atom-centered orbital (NAO) basis set.^32^

In the final step, the lowest energy candidate structures from both the MCBH and the GOFEE searches were optimized using DFT based calculations with the PBE0 hybrid functional, with a larger light-tier 1 NAO basis set. All DFT calculations were carried out with the Fritz Haber Institute Ab Initio molecular simulations package (FHI-AIMS).^33^ We note that as all considered cluster cations are formally open-shell radicals, all DFT calculations were spin-polarized. In our calculations, we assume that the experimentally prepared clusters are cold enough so that finite temperature anharmonic effects are minimal and vibrational IR spectra were calculated using the harmonic approximation. To approximately account for any small anharmonic effects and systematic functional-based effects,^34^ all frequencies were slightly downshifted by 1.5%. In addition, the calculated lines for each frequency were broadened using a Cauchy–Lorentz distribution of width 15 cm^–1^ to mirror the line widths in the experimental spectra. To provide a relative estimate of the degree of matching between an experimental spectrum and calculated spectra from possible isomers, the cosine similarity score (CSS) was calculated in all cases (see Section S4 of the Supporting Information for details).

To study the energetics of hydration, we obtained candidate low energy isomers for the following stoichiometries: Si_2_O_2_(H_2_O)_*N*_^+^, Si_2_O_4_(H_2_O)_*N*_^+^, Si_3_O_5_(H_2_O)_*N*_^+^, Si_4_O_6_(H_2_O)_*N*_^+^, and Si_4_O_8_(H_2_O)_*N*_^+^ for a range of degrees of hydration (*N* = 0–5) with the GOFEE method. The thermodynamics of the hydration reaction (eq 2) were calculated using energies and vibrations calculated using the above-noted DFT setup used for the optimizations and standard statistical thermodynamics, as detailed in previous studies.^29^ Through these calculations, we obtained the cluster free energies as a function of temperature and partial water vapor pressure.

## Results and Discussion

### Mass Spectral Characterization of Si_*x*_O_*y*_^+^ and Si_*x*_O_*y*_H_2_^+^ Clusters

**Figure 1:**
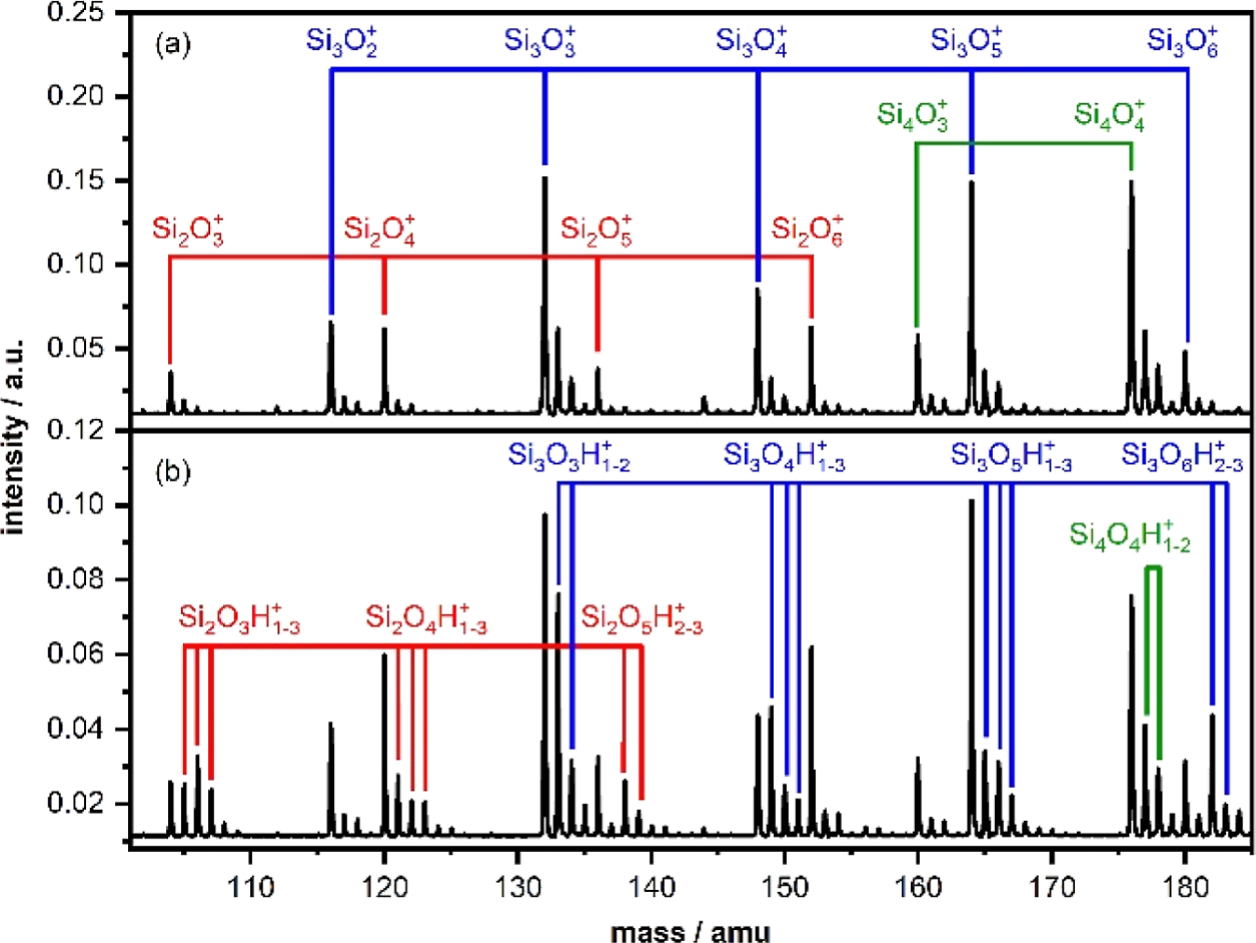
(a) Ion mass distribution of cationic silicon oxide clusters Si_*x*_O_*y*_^+^(*x* = 2–4, *y* = 2–6) produced via laser ablation of a Si target in the presence of 0.15% O_2_/He as well as (b) ion mass distribution obtained after reacting the clusters in a flow tube reactor filled with 1% H_2_O/He.

Laser ablation of a silicon rod in the presence of a 0.15% O_2_/He gas mixture leads to the production of a broad distribution of silicon oxide cations Si_*x*_O_*y*_^+^. Figure 1a shows a typical cluster distribution obtained in the mass range of 100–185 amu (for a larger mass range, see Figure S1a of the Supporting Information). Because silicon has three naturally abundant isotopes [^28^Si (92.23%), ^29^Si (4.67%), and ^30^Si (3.10%)^35^, each stoichiometry shows a progression of multiple mass peaks dominated by the ^28^Si isotopologue. In the Figure, only the ^28^Si_*x*_^16^O_*y*_^+^ stoichiometries are labeled.

The formed clusters were subsequently reacted with a 1% H_2_O/He gas mixture in a flow tube reactor resulting in product distributions, as shown in Figures 1b and S1b. In these mass spectra, we identify several products containing two hydrogen atoms (e.g., Si_2_O_3_H_2_^+^, Si_2_O_4_H_2_^+^, Si_2_O_5_H_2_^+^) or an odd number of hydrogen atoms (e.g., Si_2_O_3_H^+^, Si_2_O_3_H_3_^+^, Si_2_O_4_H^+^, Si_2_O_4_H_3_^+^), indicating dissociative water adsorption. Due to the Si natural isotope distribution and the small mass difference between O, OH, and H_2_O, the isotope distributions of the formed products typically overlap with those of the bare clusters. Moreover, upon IR irradiation, several mass channels show an increase (rather than the loss due to IR-induced dissociation), caused by fragmentation of larger species. With the aid of simulated natural isotope distributions (details are given in the Supporting Information, Figures S2–S7), we have identified that the the IR-MPD spectra of the Si_2_O_3_H_2_^+^, Si_2_O_5_H_2_^+^, Si_3_O_6_H_2_^+^, Si_4_O_7_H_2_^+^, and Si_4_O_9_H_2_^+^ clusters have no contamination due to ingrowth from larger species and have negligible isotopologue contributions from other species.

### IR-MPD Spectroscopy of Si_*x*_O_*y*_H_*z*_^+^ Clusters

IR spectra reported for cationic silicon oxide clusters show bands restricted to the 200–1100 cm^–1^ region.^18,19^ For the species under study here, one must consider the possibility of intact water adsorption, leading to bands associated with the normal modes of the water molecule. Of these, only the bending mode lies in the spectral region accessible with FELICE (1595 cm^–1^ for a free molecule,^36^ typically 1550–1700 cm^–1^ when adsorbed on a metal oxide cluster^37,38^). However, in none of the experimental spectra presented, any IR activity is observed above 1300 cm^–1^, indicating the absence of intact water molecules in the species studied. This is corroborated by the high exothermicity (<−2.4 eV) of all hydration reactions (i.e., reaction with one water molecule) of the corresponding Si_*x*_O_*y*_^+^ anhydrous clusters (see Discussion below and Supporting Information). Instead, all spectra exhibit a rich, relatively broad band structure between 600 and 1100 cm^–1^. The broadness could be attributed to the IR-MPD excitation mechanism requiring the absorption of multiple photons but also the spectral congestion due to vibrational modes involving Si–O as well as hydroxyl (−OH) groups.

**Figure 2:**
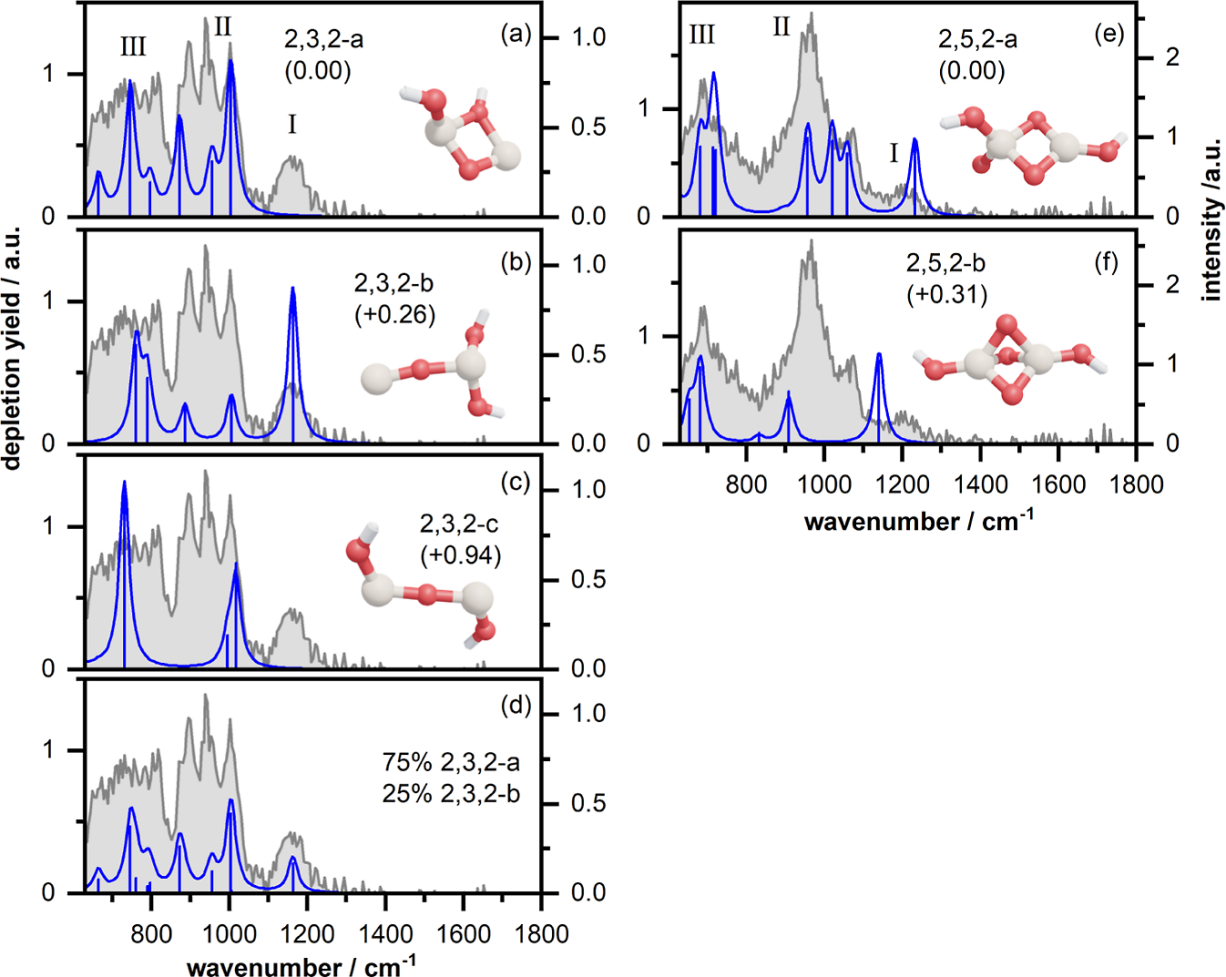
Experimental IR-MPD spectra (in gray) recorded for Si_2_O_3_H_2_^+^ (left column) and Si_2_O_5_H_2_^+^ (right column) together with the calculated vibrational spectra (in blue) of several isomeric structures (relative energies in parentheses are given in eV). The calculated stick spectra are convoluted with a Lorentz–Cauchy distribution with a broadening of 15 cm^–1^. In the structural models, Si, O, and H atoms are depicted by large light brown and smaller red and white spheres, respectively.

The left column in Figure 2 displays the IR-MPD spectrum recorded for Si_2_O_3_H_2_^+^ (shown in gray). This spectrum shows three bands: a well-separated, rather low intensity band (labeled I) centered around 1160 cm^–1^, a broad and structured band (II) between 850 and 1150 cm^–1^ indicating the presence of at least three modes, and a broad unresolved band (III) below 850 cm^–1^. The calculated lowest energy isomer of Si_2_O_3_H_2_^+^ (isomer 2,3,2-a in Figure 2a) has a rhombic Si_2_O_2_ core with one bridging hydroxyl group and a bridging oxygen atom between both Si atoms and a second terminal –OH group bound to a single silicon atom. In the chosen naming convention, 2,3,2 denotes the elemental composition *x*,*y*,*z* of Si_*x*_O_*y*_H_*z*_^+^, and *a* is the isomer sorted in order of increasing energy relative to the lowest energy isomer. Several isomeric structures only differing in the orientation of the terminal hydroxyl group were found to be almost isoenergetic (cf., Figure S8). Due to the very small structural differences, these conformers have very similar vibrational spectra (cf. Figure S9), which allows us to restrict our discussion here to the lowest energy isomer. The calculated vibrational spectrum shows several bands in the spectral region between 630 and 1000 cm^–1^. The three modes at 1004, 955, and 872 cm^–1^ are in favorable agreement with the frequencies of the structured band II, and the three modes at 797, 746, and 665 cm^–1^ might be responsible for the broad unresolved band III. The calculated 872 cm^–1^ band is about 20 cm^–1^ lower in frequency than the experimental maximum. However, the comparison of experimental intensity distributions matches less well, and isomer 2,3,2-a cannot account for band I. In contrast, a second more linear isomer (2,3,2-b in Figure 2b; further almost isoenergetic structures with similar vibrational spectra are shown in Figures S8 and S9) shows a mode at 1163 cm^–1^, corresponding to the stretching motion of the terminal Si–O unit, which agrees with band I. For this isomer, four other bands are predicted: two at 790 and 761 cm^–1^ falling in the spectral window of the unresolved band III and two at 1005 and 886 cm^–1^ matching the right and left maxima of band II. However, no mode matching the middle band is offered. Thus, the observation of band I points to the presence of isomer 2,3,2-b, but the full structure of band II is only satisfied if isomer 2,3,2-a is also present. A third linear isomer (2,3,2-c in Figure 2c), with +0.94 eV considerably higher in energy, has a band around 1000 cm^–1^ and one at 731 cm^–1^; its presence in the molecular beam cannot be excluded, but it clearly cannot explain band I or the structure of bands II and III. If we compare the CSS for the individual isomers, we find the highest value of 0.89 for isomer 2,3,2-a, whereas both other isomers score below 0.7. To account for band I, we present the spectrum for an empirical mixture of 75% 2,3,2-a and 25% 2,3,2-b, which leads to an improved CSS of 0.91 (cf. Figure 2d), where we have disregarded isomer 2,3,2-c based on its relatively high energy. To summarize, the coexistence of isomers 2,3,2-a and 2,3,2-b can satisfactorily explain all features of the IR-MPD spectrum.

The spectrum observed for Si_2_O_5_H_2_^+^ (right column of Figure 2) is significantly different from that of Si_2_O_3_H_2_^+^: band I is less intense and blue-shifted to around 1190 cm^–1^, band II comprises an intense part centered around 960 cm^–1^ with a sideband around 1070 cm^–1^ (instead of three bands as for Si_2_O_3_H_2_^+^), and band III is less broadened. The calculated spectrum of the lowest energy isomer 2,5,2-a (cf. Figure 2e; for other almost isoenergetic isomers, cf. Figures S8 and S10) shows modes with frequencies that roughly agree with those of bands I, II, and III. With respect to intensities, the mode predicted at 1232 cm^–1^ is somewhat more intense than that observed in band I. Conversely, the high intensity of the main maximum of the two maxima observed in band II is higher than in the computed spectrum. Here, this mismatch could be resolved by assuming a greater overlap of the predicted 1020 and 1059 cm^–1^ peaks, perhaps being closer in experiment than predicted and merging into one main peak. This would help rationalize that band II for Si_2_O_5_H_2_^+^ is twice as intense as that for Si_2_O_3_H_2_^+^. Despite these concerns, the match with the experiment for 2,5,2-a is superior than that for a second, more symmetric, and higher energy isomer 2,5,2-b (cf. Figures 2f, S8 and S10). Therefore, we conclude that the IR-MPD spectrum is best described by the lowest energy isomer 2,5,2-a. Due to the energetic degeneracy and the similarity of the lR spectra, the coexistence of isomers with rotated OH groups (cf. Figure S10) is likely. The assignment of 2,5,2-a to the experimental spectrum is also supported by its relatively high CSS (0.81) compared to 2,5,2-b (0.58).

**Figure 3:**
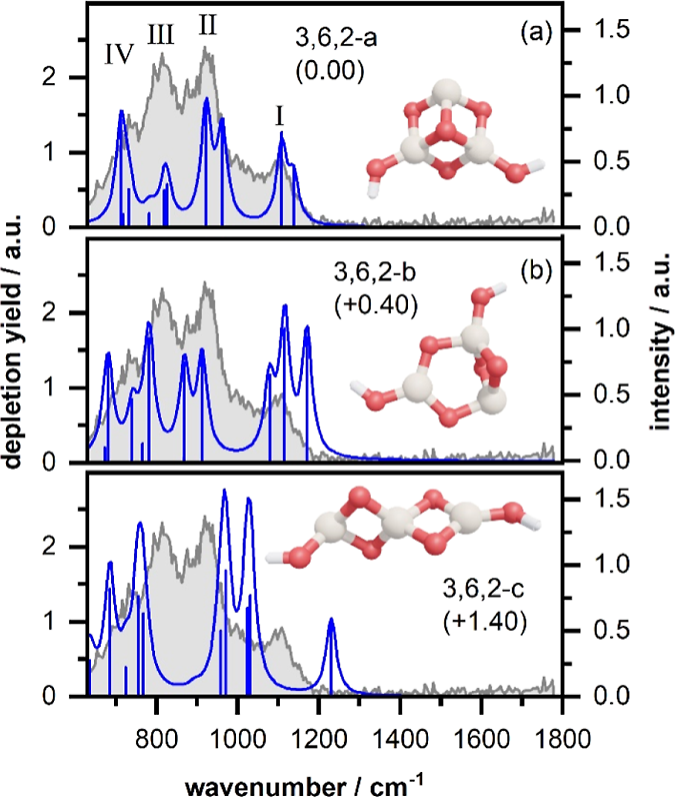
Experimental IR-MPD spectra (in gray) recorded for Si_3_O_6_H_2_^+^ together with calculated vibrational spectra (in blue) of three isomers (relative energies in parentheses are given in eV). For details, see caption of Figure 2.

The IR-MPD spectrum of Si_3_O_6_H_2_^+^ exhibits three rather broad bands centered at 1096, 921, and 815 cm^–1^, the latter with a shoulder at around 700 cm^–1^ (see Figure 3). The calculated lowest energy structure is shown in Figure 3a, and two more almost isoenergetic isomers differing only by the orientation of the hydroxyl groups are shown in Figure S8. The calculated IR spectrum for 3,6,2-a shows four groups of vibrations that agree fairly well with the IR-MPD spectrum. The occurrence of several modes in the spectral region between 650 and 900 cm^–1^ can also explain the rather broad bands observed in the experiment. If there is a weak point in the comparison, it could be the strength of the observed band III, but this could be due to a cooperative effect in the IR-MPD excitation process. The assignment to isomer 3,6,2-a is further confirmed by the calculated spectra of two higher energy isomers (isomer 3,6,2-b in Figure 3b and isomer 3,6,2-c in Figure 3c). Both higher energy isomers show features that are clearly in disagreement with the IR-MPD spectrum, in particular, in the spectral region of band I. The CSS score of 3,6,2-a (0.88) is also higher than that of the other two isomers (0.80 for 3,6,2-b and 0.68 for 3,6,2-c).

**Figure 4:**
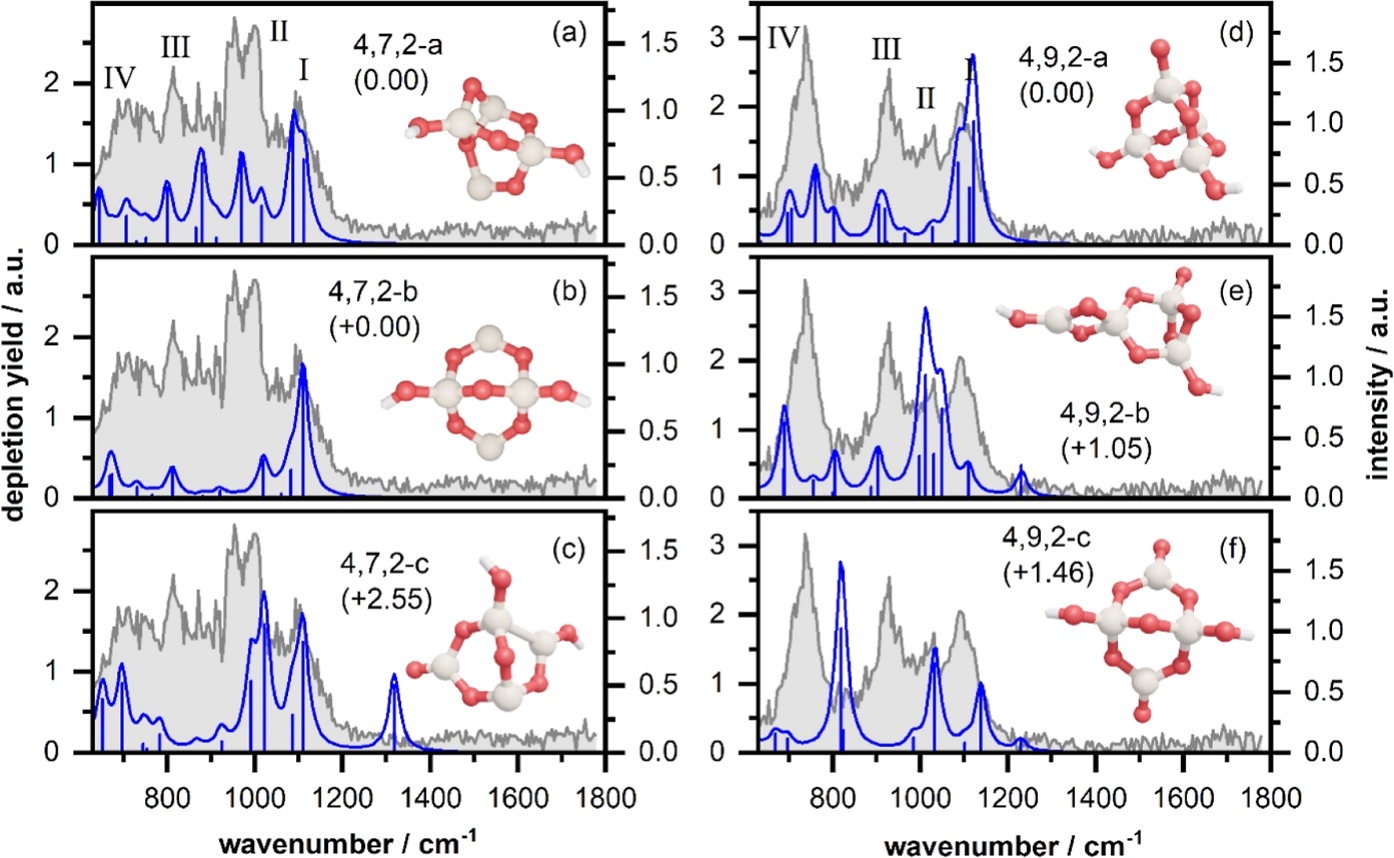
Experimental IR-MPD spectra (in gray) recorded for Si_4_O_7_H_2_^+^ (left column) and Si_4_O_9_H_2_^+^ (right column) together with the calculated vibrational spectra (in blue) of several isomeric structures (relative energies in parentheses are given in eV). For details, see caption of Figure 2.

Finally, we studied Si_4_O_7_H_2_^+^ (left column of Figure 4) and Si_4_O_9_H_2_^+^ (right column of Figure 4). The IR-MPD spectra of both clusters show four rather broad bands between 630 and 1200 cm^–1^. For Si_4_O_7_H_2_^+^, we found two isoenergetic structures. The calculated vibrational spectrum of isomer 4,7,2-a (cf. Figure 4a; cf. Figures S8 and S13 for more isoenergetic isomers) shows two modes at 1112 and 1089 cm^–1^ in agreement with band I (centered at 1095 cm^–1^), two modes at 1015 and 970 cm^–1^ in agreement with the double band II (centered at 991 and 956 cm^–1^), and a mode at 878 cm^–1^, although blue-shifted, potentially describing band III (centered at 817 cm^–1^), as well as several modes below 800 cm^–1^ which are reflected in band IV. Thus, although the intensities of the modes at 1015 and 970 cm^–1^ appear to be a little underestimated, isomer 4,7,2-a can describe all the observed experimental features.

A second, more open isomer 4,7,2-b (Figure 4b; cf. Figures S8 and S13 for more isomers) is isoenergetic to 4,7,2-a and shows numerous modes between 630 and 1200 cm^–1^. Although all modes fall in the spectral window of the absorptions observed, the overall match is poorer, notably for band III. A third, high energy isomer 4,7,2-c (Figure 4c) can certainly be ruled out, especially because of the lack of the predicted band just above 1300 cm^–1^. We conclude that the IR-MPD spectrum is best described by isomer 4,7,2-a, but contributions from isomer 4,7,2-b could certainly exist. The relatively large CSS of 4,7,2-a (0.9) compared to those of the other two isomers (<0.74) tends to confirm its presence in the experiment.

The IR-MPD spectrum of Si_4_O_9_H_2_^+^ is shown in the right column of Figure 4. The lowest energy isomer (4,9,2-a in Figure 4d; more similar isomers are given in Figures S8 and S12) has a compact cage-like structure. Due to the increased cluster size, many vibrational modes are predicted in the spectral region below 1200 cm^–1^. Several modes at around 900 and 1100 cm^–1^ are potentially in agreement with bands I and III, respectively, and the modes predicted below 800 cm^–1^ can all contribute to the intense band IV. Even the experimental band II at 1022 cm^–1^ is consistent with the predicted mode at 1027 cm^–1^. Although the match between the experimental and theoretical spectra is not perfect, notably for the predicted intensities for bands II and III, isomer 4,9,2-a is consistent with the overall structure of the IR-MPD spectrum. Further isomers found are at least 1 eV higher in energy (isomers 4,9,2-b and 4,9,2-c in Figure 4e,f) and therefore are questionable as contributors. As for all the cases considered, we again find that the lowest energy isomer has a larger CSS (0.81) than competing higher energy isomers (<0.58).

### Structural Evolution of Hydroxylated Silicon Oxide Clusters

The most stable structures of small neutral stoichiometric (SiO)_*n*_ (*n* = 2–4) clusters are predicted to have a ring structure.^39−41^ Conversely, stable neutral oxygen rich (SiO)_*n*_O_*m*_ (*n* = 2–4, *m* = 1–3) clusters tend to form chains of rhombic Si_2_O_2_ rings, each sharing a Si atom, with the remaining oxygen atoms terminally coordinated to the Si atoms at the ends of the chain.^40,41^ Only for the lowest energy Si_4_O_5_ isomer, we do see a deviation from this tendency, with a structure that links a Si_3_O_3_ ring and a Si_2_O_2_ ring via a shared Si atom.^40,41^ Cationic clusters with the corresponding range of compositions and identified by their IR-MPD spectra and chemical modeling also exhibit such rings in addition to Si–Si bonds.^18,19^ Small strained rings, terminal oxygen atoms, and direct bonding between Si atoms are markedly different from the networks of tetrahedral SiO_4_ units observed in bulk silica. For neutral clusters, the hydroxylation via dissociative water adsorption is likely to convert such sites to more relaxed four-coordinated centers.^28,29^ Considering this, we will now discuss the structures of the cationic hydroxylated silicon oxide clusters.

Both Si_2_O_2_^+^ and Si_2_O_4_^+^ were theoretically predicted to have a rhombus-like geometry with the additional two oxygen atoms of Si_2_O_4_^+^ terminally coordinated to the Si atoms.^10,42^ Addition of a H_2_O unit, yielding Si_2_O_3_H_2_^+^ and Si_2_O_5_H_2_^+^, retains the rhombic cluster core (see Figures 2a,e). While for Si_2_O_3_H_2_^+^, a 3-fold coordination of the Si atoms cannot be exceeded, a 4-fold coordinated SiO_3_(OH) is observed in Si_2_O_5_H_2_^+^. Besides these structures, we also find two rather unexpected geometries which are only about 0.3 eV higher in energy (see Figure 2b,f): a linear (OH)_2_Si–O–Si structure for Si_2_O_3_H_2_^+^, which seems to represent a step toward cluster dissolution, and a structure with two face-sharing tetrahedrons for Si_2_O_5_H_2_^+^. This latter structure is particularly rare due to the very sterically strained congested triple oxygen bridge,^43^ and as far as we are aware, this has never been reported in the context of Si–O bonded structures.

The structures of cationic Si_3_O_5,6_^+^ clusters have not been reported so far. However, Si_3_O_4_^+^ was experimentally found to be a chain of two Si_2_O_2_ rhombuses^20^ in agreement with the cluster core of the neutral Si_3_O_5,6_.^40,41^ We find here that the addition of one H_2_O unit to Si_3_O_5_^+^ (yielding Si_3_O_6_H_2_^+^) changes the structure completely to an open cube with one vertex missing with two SiO_3_(OH) units (see Figure 3a). This three-dimensional structure allows the maximal coordination of the Si atoms (two atoms are 4-fold and one atom 3-fold coordinated), which is not achieved in the bare cluster Si_3_O_6_ (one atom 4-fold and two atoms 3-fold coordinated).

The structures of Si_4_O_4_^+^ and Si_4_O_5_^+^ were reported in a previous cluster beam IR-MPD study.^19^ The former consists of a Si_3_O_2_ ring (exhibiting a Si–Si bond) attached to a rhombic Si_2_O_2_ ring via a shared Si atom, whereas the latter consists of Si_2_O_2_ and Si_3_O_3_ units with a shared Si atom. A similar structure was predicted for neutral Si_4_O_5_ showing that also in this case, the charge state does not significantly influence the geometry.^40,41^ The higher oxidized clusters Si_4_O_6,7,8_ are predicted to consist of chains of Si_2_O_2_ rhombuses.^40,41^ Our calculations for the tetra-silicon species, as for the trisilicon species, indicate a considerable structural change upon adsorption of one H_2_O unit. For Si_4_O_7_H_2_^+^, we found two almost isoenergetic structures, one based on a Si_4_O_4_ ring with an additional Si–O–Si bridge (cf. Figure 4b) and one more compact structure consisting of a Si_2_O_2_ ring with Si_3_O_3_ rings (cf. Figure 4a), both containing two SiO_3_(OH) units. In both structures, two of the four Si atoms are 4-fold coordinated. Interestingly, a Si_4_O_4_ ring-based structure was found to be 0.36 eV less stable than the Si_2_O_2_/Si_3_O_3_ rings for the bare Si_4_O_5_^+^ cluster,^19^ indicating that this structure becomes stabilized upon addition of the hydroxyl groups. In the Si_4_O_9_H_2_^+^ cluster, the four Si atoms form a tetrahedron with all edges being O-bridged and the remaining oxygen atoms bound as terminal Si=O and Si–OH groups. In this isomer, three of the four Si atoms are 4-fold coordinated. A similar 3D structure has theoretically been predicted for the anionic Si_4_O_9_H_2_^–^, whereas for Si_4_O_7_H_2_^–^, a structure based on a chain of Si_2_O_2_ rings has been found.^16^

To summarize, for the small disilicon oxide clusters, the rhombic cluster core is retained upon addition of an H_2_O unit, whereas the tri- and tetra-silicon oxide clusters undergo a significant structural transformation with respect to the corresponding bare cationic clusters. Similar effects have previously been predicted for neutral silica clusters,^28,29^ where such large structural changes can also be induced by the dissociative addition of a single H_2_O unit leading to final cluster geometries which are better able to maximize the coordination of the Si atoms.

### Astronomical Relevance

#### Astrochemistry

SiO molecules are abundantly formed in the envelopes of evolved O-rich stars, where they are assumed to be an essential precursor for silicate dust formation.^44^ Here, reactions at low pressures (0.1–0.001 Pa) and high temperatures (1000–1200 K) involving SiO, OH/H_2_O, and Mg are thought to play a role in the initial steps of silicate dust formation.^45−47^ Here, however, any such produced neutral Si_*x*_O_*y*_H_*z*_ species would likely be short-lived nucleation intermediates. Once formed, silicate dust grains leave their circumstellar birthpace and enter the ISM where energetic processing (e.g., by supernovae shocks) leads to sputtering of Si atoms and/or Si-bearing material, and subsequent reformation of SiO molecules.^48^ Despite this possible SiO formation route, recent detection of highly abundant SiO molecules in the diffuse and translucent ISM suggests that shocked dust may not be the primary source of interstellar SiO.^49^ The high abundance of SiO is, however, consistent with the expected high production of SiO predicted by models of gas phase silicon chemistry in translucent^50^ and dense molecular clouds.^51^ Here, the efficiency of SiO formation appears to be reliant on the proposed presence of a number of positively ionized species (e.g., Si^+^, SiO^+^, and SiOH^+^) and their reactions with H_2_, H_2_O, and OH.^51^ The potential presence of these species in the ISM is also supported by experiments showing that hydrogen acts to stabilize cationic Si_*x*_O_*y*_H_2_^+^ clusters.^13^ Subsequent condensation of neutral SiO molecules into (SiO)_N_ oligomers should also be possible at relatively cold conditions in such environments.^52,53^ Overall, translucent and dense clouds seem to possess all of the necessary conditions and ingredients for producing a range of stable cationic Si_*x*_O_*y*_H_*z*_^+^ clusters.

Considering the uncertainty in dust grain destruction rates versus the rate of production in known sources (e.g., AGB stars), it is possible that additional dust formation in the ISM via neutral and charged Si_*x*_O_*y*_H_*z*_ species may also be occurring.^52,54^ As our species are charged, this could also potentially enhance their role as seeds for nucleation.^55^ We further note that Si_*x*_O_*y*_H_*z*_^+^ clusters are radical cationic species that have been shown to be capable of activating methane^13,42^ and thus could also play a role in catalyzing carbon-based interstellar chemistry. In the following, we will evaluate the thermodynamics of the hydration reaction for the case of Si_2_O_2_^+^ in order to assess the feasibility of Si_*x*_O_*y*_H_*z*_^+^ formation in both our experiment and in the ISM.

In our cluster beam experiment, the cluster formation path is thought to follow the production of Si_*x*_O_*y*_^+^ species and subsequent reaction with H_2_O. For the smallest cluster observed in the current study, this would entail the following hydration reaction (where all energies below are 0 K reaction enthalpies derived from the DFT-calculated total energies of the species involved)
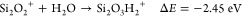
1

**Figure 5:**
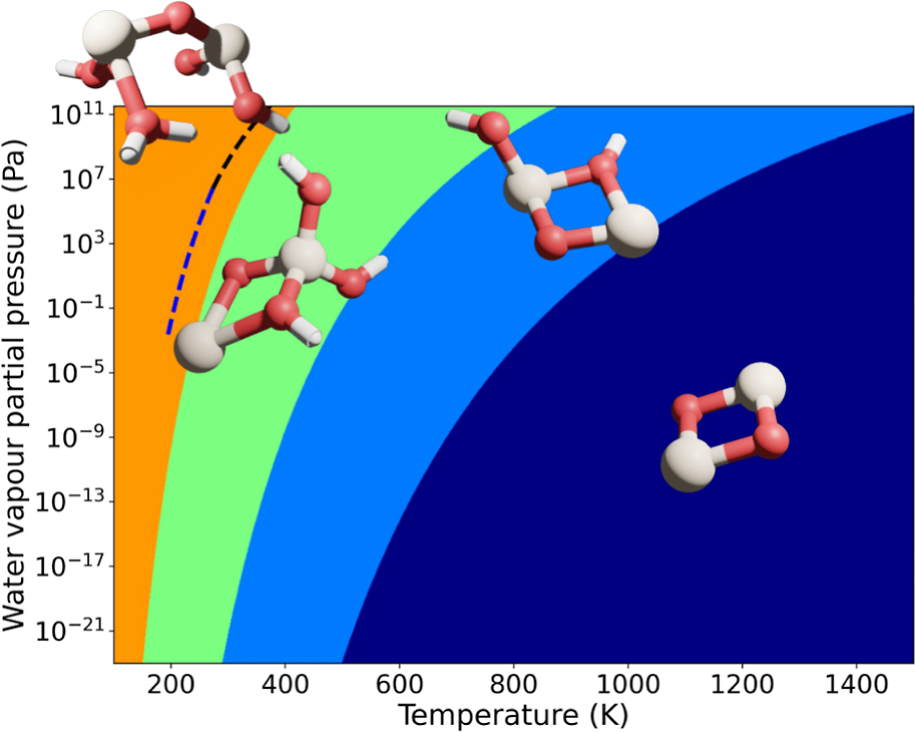
Calculated water vapor partial pressure versus temperature free energy phase diagram for Si_2_O_2_(H_2_O)_*x*_^+^ (*x* = 0–3) formation. Inset structures indicate the lowest free energy isomer for each colored region, where the degree of hydration increases from left to right. The dashed curve indicates the water condensation line. Calculated free energy phase diagrams for other cluster sizes are included in the Supporting Information.

Assuming a prior reaction of the type
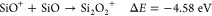
2the exothermic hydration reaction 1 could also potentially occur in moderately dense regions of the ISM where water molecules are available reactants. To assess the thermodynamic favorability of Si_2_O_2_^+^ hydration more generally, we have calculated the Gibbs free energy for reaction 1 under various conditions of temperature and partial water vapor pressure (see Figure 5). From the calculated phase diagram, we clearly see that dissociative addition of two or three water molecules to the Si_2_O_2_^+^ cluster is predicted over a very wide range of partial pressures of H_2_O for temperatures less than 400 K (orange region in Figure 5). For the partial pressure of water vapor expected in our experiments (of the order 10 kPa), it is predicted that the temperature should be above 500 K to favor only the addition of a single H_2_O molecule for the observed Si_2_O_3_H^+^ cluster. In our experiments, soon after ablation, the conditions are thought to be around at room temperature and very likely significantly lower than 500 K. Several possibilities could explain this seeming discrepancy. First, the H_2_O addition reactions could be exothermic but possess significant barriers, limiting the hydration degree. This may be compared to the reaction of a single water molecule with a neutral Si_2_O_2_ cluster which is predicted to be both very exothermic and inhibited by a kinetic barrier.^46,47^ Perhaps, a more likely reason for the addition of only limited hydroxylation is the rather short (100 ms) time that the clusters are exposed to water in the flow tube reactor meaning that thermodynamic equilibrium (assumed in Figure 5) is not achieved. We note that this experimental condition can be controlled to some extent, thus allowing for mimicking the degree of hydroxylation expected in different astrophysical environments.

We note that although the experimentally observed species are most likely formed by H_2_O addition, we can also consider alternative reaction pathways for their formation in the ISM. Formation of Si_2_O_3_H^+^ has also been observed in experiments based on sputtering of pure silicon targets where the incorporation of hydrogen is assumed to come from surface hydroxyl groups.^13^ Below, we thus tentatively suggest an alternative formation pathway via the exothermic reactions 3 and 4. We note that such a pathway likely involves species in translucent clouds^50^ (see above) and thus could also be of potential relevance to interstellar silicon chemistry.
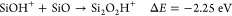
3
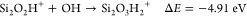
4

#### IR Spectroscopy

Other than trying to predict the likelihood of interstellar formation of our species, we can point to identifying spectroscopic features that could be detected with sufficiently high-resolution IR observations (e.g., by JWST). For all cluster sizes, we observe a characteristic band between 1090 and 1200 cm^–1^ (labeled band I in Figures 2−4). The position of this band shows some cluster size dependence (1159 cm^–1^ for Si_2_O_3_H_2_^+^, 1197 cm^–1^ for Si_2_O_5_H_2_^+^,1098 cm^–1^ for Si_3_O_6_H_2_^+^, 1094 cm^–1^ for Si_4_O_7_H_2_^+^, and 1092 cm^–1^ for Si_4_O_9_H_2_^+^) but remains well separated from the bands observed below 1050 cm^–1^.

In the case of Si_2_O_3_H_2_^+^, band I can be explained by the stretching motion of the terminal −O–Si unit of isomer 2,3,2-b1. Small cationic magnesium silicate cluster isomers also exhibit similarly high frequency stretching modes, which are associated with non-hydrogen-containing terminal groups attached to constrained SiOMgO rings.^56^ For all other clusters studied here, band I is associated with the Si–OH stretching motion (νSi–OH) of isolated silanol (SiOH) groups, with an average frequency of 1092 cm^–1^. We note that isomer 2,3,2-b1 only possesses a geminal silanol group. On bulk silica surfaces, νSi–OH of isolated silanol groups has experimentally been determined to be 975 ± 5 cm^–1^.^57^ This mode lies in the spectral region of typical bulk νSi–O vibrations, which makes its assignment difficult.^58^ In small silanol-containing molecules, νSi–OH has been calculated to lie in the range between 790 and 1030 cm^–1^ and strongly depends on substituents close to the Si atom (e.g., electron-withdrawing groups result in a higher wavenumber than electron-donating groups).^59^ For such small systems, νSi–OH is often coupled with vibrations of surrounding groups.

Compared with hydroxylated bulk silica and small inorganic compounds, we thus observe a considerably blue-shifted νSi–OH for isolated silanols on our small weakly hydroxylated cationic clusters. This blue shift is also considerably larger than expected based on the small size (i.e., smaller reduced mass compared to bulk silica) of the clusters, indicating a fundamental physical (chemical) difference instead of a simple size effect. Such a difference can lie in the Si–OH bond strength. The Si–OH bond length in minerals and inorganic compounds tends to decrease with the number of bridging O atoms bound to the silicon atom of the silanol group ranging from an average value of ∼1.69 Å for compounds without bridging oxygen atoms to ∼1.60 Å for tetrahedra with three bridging O atoms.^60,61^ In contrast, the Si–OH bond lengths in the investigated clusters here range between 1.55 and 1.58 Å. These anomalously small Si–OH bond lengths and their particularly high frequency νSi–OH vibrations thus seem characteristic of small weakly hydroxylated cationic Si_*x*_O_*y*_H_*z*_^+^ clusters. In Table 1, we show this effect by comparing the Si–O bond lengths and the νSi–OH frequencies in the isolated silanols of both neutral and cationic versions of our Si_*x*_O_*y*_H_*z*_ isomers. Typically, the blue shift of νSi–OH upon positively charging our clusters is around +40–50 cm^–1^, which is associated with an average 0.03 Å decrease in the Si–O bond length. From our small sample of clusters, this effect does not seem to be strongly related to cluster size. However, calculations on progressive hydration of neutral silica clusters of different sizes suggests that increasing the degree of hydroxylation could also augment the blue shifting of νSi–OH for isolated silanols.^29^

**Table 1:** Shift in Si–OH Bond Lengths and νSi–OH Vibrational Frequencies/Wavelengths between Neutral and Cationic Clusters for a Range of Stoichiometries^a^

	d(Si–OH) cation (Å)	d(Si–OH) neutral (Å)	Δ[d(Si–OH)] (Å)	νSi–OH cation (cm^–^^1^/μm)	νSi–OH neutral (cm^–^^1^/μm)	Δ(νSi–OH) (cm^–^^1^/μm)
Si_2_O_5_H_2_	1.59	1.64	–0.05	1219/8.20	1180/8.47	+39/–0.27
	1.56	1.59	–0.03			
Si_3_O_6_H_2_	1.58	1.61	–0.03	1126/8.88	1042/9.59	+84/–0.71
				1097/9.11	1016/9.84	+81/–0.73
Si_4_O_7_H_2_	1.59	1.62	–0.03	1079/9.26	1035/9.66	+44/–0.40
				1041/9.60	1020/9.80	+21/–0.20
Si_4_O_9_H_2_	1.58	1.60	–0.02	1108/9.02	1066/9.38	+42/–0.36
				1100/9.09	1061/9.42	+39/–0.33
average	1.58	1.61	–0.03	1110/9.00	1060/9.43	+50/–0.43

a The lowest energy species was used to calculate the structures and IR spectra in each case. Note that in the case of Si_3_O_6_H_2_, the structures of the lowest energy neutral and cationic isomers are different.

**Figure 6:**
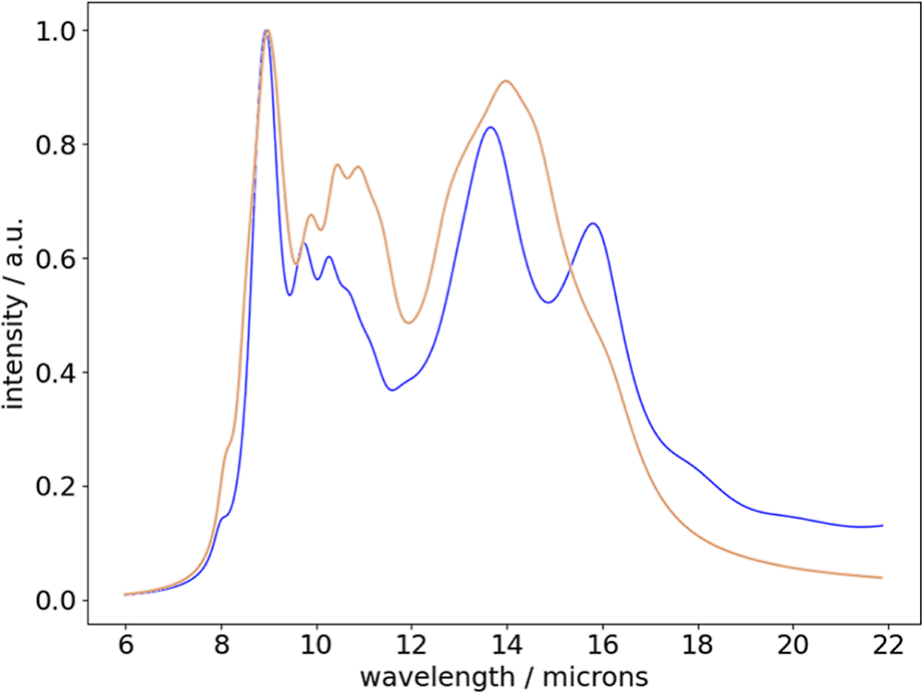
Combined IR spectrum obtained from summing calculated IR spectra from low energy isomers from all distinct cluster stoichiometries shown in Figures 2–4. The blue line corresponds to only the lowest energy isomers, and the orange line corresponds to all isomers within 0.5 eV of the lowest energy isomers.

In astronomical IR observations, a broad feature ranging approximately between 800 and 1200 cm^–1^ and peaked at about 1030 cm^–1^ (9.7 μm) is interpreted to correspond to different asymmetric and symmetric Si–O stretching modes of the SiO_4_ tetrahedra in silicate cosmic dust.^62^ In amorphous silicates, Si–O stretching frequencies in the higher frequency region (around 1110 cm^–1^/9 μm) are typically attributed to Si–O stretching modes in a segregated pure silica components.^63^ We note that, although pure bulk silica has been identified in IR observations of protoplanetary disks, it is not thought to be a significant dust component in the diffuse ISM due to the absence of other signature IR peaks (e.g., at 9, 12.6, 16, and 20 μm).^64^ As mentioned above, silanol groups on bulk silica exhibit modes around 975 cm^–1^ (10.2 μm), which are considerably lower in frequency than the corresponding bulk Si–O stretching modes. In our Si_*x*_O_*y*_H_*z*_^+^ clusters, we see an inversion of this tendency, whereby the νSi–OH modes have higher frequencies (1090–1197 cm^–1^) than Si–O stretching modes in nonterminal parts of the clusters. Furthermore, these νSi–OH modes lie toward the extreme high frequency tail of those expected for a typical silicate dust IR feature where the intensity usually quickly falls to zero. We thus suggest that populations of Si_*x*_O_*y*_H_*z*_^+^ clusters could tend to lead to a longer and more intense high frequency (low wavelength) tail of the 9.7 μm Si–O stretching band, or even distinguishable IR peaks around 9 μm in this spectral region (see Figure 6). The positive charge and low hydroxylation of these species would probably make them more likely to be observed in the diffuse/translucent regions of the ISM, although their potential presence in other environments, such as denser clouds, protoplanetary disks, and exoplanetary atmospheres, cannot be ruled out.

Other IR active modes of our Si_*x*_O_*y*_H_*z*_^+^ clusters between 600 and 1000 cm^–1^ would tend to overlap with the higher wavelength part of the Si–O stretching silicate feature and contribute to the low wavelength side of the 18 μm signature (silicate O–Si–O bending mode). In this way, the effect of a population of Si_*x*_O_*y*_H_*z*_^+^ isomers could be expected to have a similar observational impact on the silicate feature as predicted for populations of nanosilicate clusters.^65^ In Figure 6, we show the combined IR spectrum formed from summing the calculated IR spectra from low energy isomers from all studied cluster stoichiometries, clearly showing a sharp peak centered around 8.9–9.0 μm and broader features in the 13–17 μm range.

## Conclusions

We produced small silicon oxide clusters via laser ablation of a silicon target in the presence of a gas pulse containing molecular oxygen seeded in helium. Subsequent reaction of the clusters with water vapor in a flow tube reactor resulted in the formation of Si_*x*_O_*y*_H_*z*_^+^ cluster species, which indicates dissociative water adsorption. Water dissociation is confirmed by IR-MPD spectroscopy of selected hydroxylated clusters, Si_*x*_O_*y*_H_2_^+^ with *x* = 2–4, lacking any signals in the water bending mode region. Further structural and vibrational characterization was achieved by comparing the experimental IR-MPD spectra with IR spectra obtained by DFT calculations. While small bare silicon oxide clusters typically contain small, strained rings, terminal oxygen atoms, and direct bonding between Si atoms, hydroxylation appears to induce a considerable structural change of the tri- and tetra-silicon oxide clusters leading to more relaxed three-dimensional geometries with higher coordination of the Si atoms and the tendency to form SiO_3_(OH) units. Only the disilicon oxide clusters retain the planar rhombic geometry of the bare cluster. However, rather unexpected geometries with higher Si atom coordination were found to be only about 0.3 eV higher in energy.

Based on the current knowledge of silicon-based astrochemistry, weakly hydroxylated cationic Si_*x*_O_*y*_H_*z*_^+^ (*z* < *y*) clusters might also be formed in the diffuse/translucent ISM. Here, and in other astrophysical environments, these clusters might serve as seeds for dust nucleation and may also play a role as catalysts for carbon-based interstellar chemistry. In the future, such species might be identified by JWST based on their specific signatures in the IR spectral region. In particular, we found that such small clusters show a characteristic IR band corresponding to the Si–OH stretching vibration, which has a higher frequency than typical silicate Si–O stretching modes and is in a range usually associated with the presence of pure silica. In our case, this relatively high frequency mode is associated with an anomalously short Si–OH bond length in the small cationic clusters. We suggest that this band could lead to a longer and more intense high frequency tail in the 9.7 μm silicate dust Si–O stretching feature or even discernible features around 9 μm in this spectral region. The potential presence of Si_*x*_O_*y*_H_*z*_^+^ species in the diffuse/translucent ISM could thus be confirmed by these signature IR features using JWST observations.

## Supporting Information Available

Experimental and theoretical methods, further isomeric structures and their vibrational spectra, CSS values, and Cartesian coordinates of the lowest energy cluster isomers (PDF)
