# Supplementary material for: Gas-Phase Production of Hydroxylated Silicon Oxide Cluster Cations: Structure, Infrared Spectroscopy, and Astronomical Relevance
Source: ACS Earth Space Chem. 2024 May 9;8(6):1154–64. doi: 10.1021/acsearthspacechem.3c00346 (PMC11194846; doi:10.1021/acsearthspacechem.3c00346)
Supplement: Supplementary file 1 — sp3c00346_si_001.pdf [file sp3c00346_si_001.pdf]

Supporting Information for:

Gas-phase production of hydroxylated silicon oxide cluster cations: structure, infrared spectroscopy and astronomical relevance

Andreu A. de Donato[a], Bianca-Andreea Ghejan[b], Joost M. Bakker[c], Thorsten M. Bernhardt[b], Stefan T. Bromley[a,d], Sandra M. Lang[b*]*

[a] Departament de Ciència de Materials i Química Física & Institut de Química Teòrica i Computacional (IQTCUB), Universitat de Barcelona, c/ Martí i Franquès 1-11, 08028 Barcelona, Spain

[b] Institute of Surface Chemistry and Catalysis, University of Ulm, 89069 Ulm, Germany.

[c] Radboud University, Institute of Molecules and Materials, FELIX Laboratory, 6525 ED Nijmegen, The Netherlands

[d] Institució Catalana de Recerca i Estudis Avançats (ICREA), Passeig Lluís Companys 23, E-08010 Barcelona, Spain

Contents:

Pages S2-S5: **Section S1 - Production and mass assignment of $Si_xO_yH_2^+$ complexes**

Page S6: **Section S2 - Isomeric structures of $Si_xH_yH_2^+$ clusters**

Pages S7-S9: **Section S3 - Additional vibrational spectra**

Page S10: **Section S4 - Cosine similarity scores (CSS)**

Pages S11-S12: **Section S5 - Calculated free energy phase diagrams**

Pages S13-S14: **Section S6 – Cartesian coordinates of lowest energy cluster isomers**

S1 - Production and mass assignment of $Si_xO_yH_2^+$ complexes

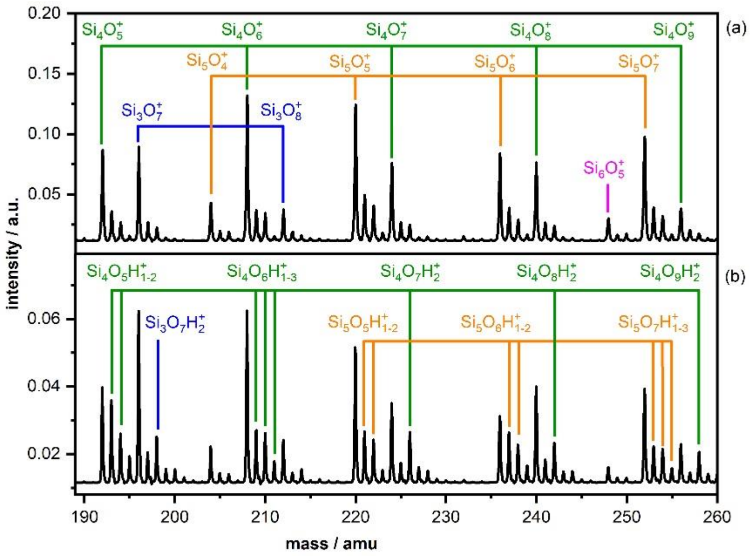

Figure S1. Ion mass distribution of cationic silicon oxide clusters $Si_xO_y^+$(x=2-4, y=2-6) produced via laser ablation of a Si target in the presence 0.15% O_2/He as well as (b) ion mass distribution obtained after reacting the clusters in a flow tube reactor filled with 1% H_2O/He. Compared to Figure 1 of the main manuscript an extended mass range is shown.

Figures 1a and S1a show that the natural isotope distribution of silicon (^{28}Si : ^{29}Si : ^{30}Si = 92.23% : 4.67% : 3.10%,)[1] leads to several peaks in the mass spectrum for each cluster size (one intense peak and several low intensity peaks). Due to the small mass difference between O, OH, and H_2O, the isotope distributions of the products formed upon introduction of water (cf. Figures 1b and S1b) typically overlap with the isotope distributions of the bare clusters. Thus, a certain mass peak can have contributions from the bare cluster as well as the water product (e.g. the peak at 226 amu has contributions from $^{28}Si_2{}^{29}Si_2O_7^+$, $^{28}Si_2{}^{29}Si^{30}SiO_7^+$, and $Si_4O_7H_2^+$), which might lead to features in the IR-MPD spectra not arising from the water product but from the bare cluster. To avoid such a contamination of the IR-MPD spectra we have simulated the isotope distributions of the bare clusters and the water products and estimated the potential contamination. For subsequent evaluation, only water products were considered for which the effect of bare clusters or other water products on the IR-MPD spectrum was negligible.

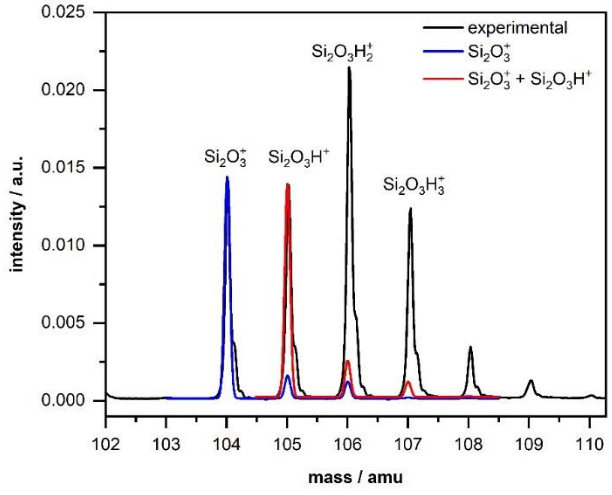

Figure S2. Experimentally obtained mass spectrum (black curves) in the mass range of $Si_2O_3H_2^+$ (m = 106 amu) together with simulated isotope distributions for the bare cluster $Si_2O_3^+$ as well as the water product $Si_2O_3H^+$.

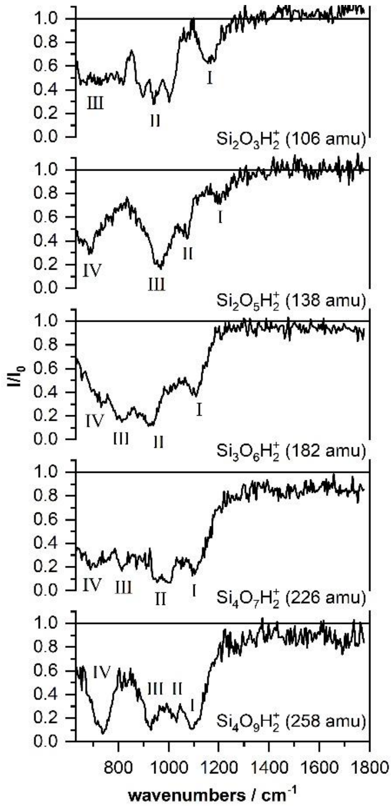

Figure S3. IR-MPD depletion spectra. To obtain the depletion yield spectra shown in the main text, these depletion spectra were corrected by the wavenumber dependent macropulse energy $P(\tilde{\nu})$ (cf. methods section).

Figure S2 shows the experimentally obtained mass spectrum (black curve) in the spectral region of $Si_2O_3^+$ (104 amu). The red curve represents the isotope distribution of $Si_2O_3^+$ calculated on basis of the natural Si isotope abundance. Upon reaction with water the mass peaks at 105, 106, and 107 amu grow (cf. Figure 1 of the main text), which correspond to the products $Si_2O_3H^+$, $Si_2O_3H_2^+$, and $Si_2O_3H_3^+$. The green curve represents the sum of the calculated isotope distributions of $Si_2O_3^+$ and $Si_2O_3H^+$. This shows that the mass peak at 106 amu, corresponding to $Si_2O_3H_2^+$ has a small contribution (red curve at 106 amu) of the bare cluster $^{29}Si_2O_3^+/^{28}Si^{30}SiO_3^+$ as well as a small contribution (difference between red and green curve at 106 amu) of $^{28}Si^{29}SiO_3^+$. Based on this we estimate a contribution of about 12% of $Si_2O_3^+/Si_2O_3H^+$ to the mass

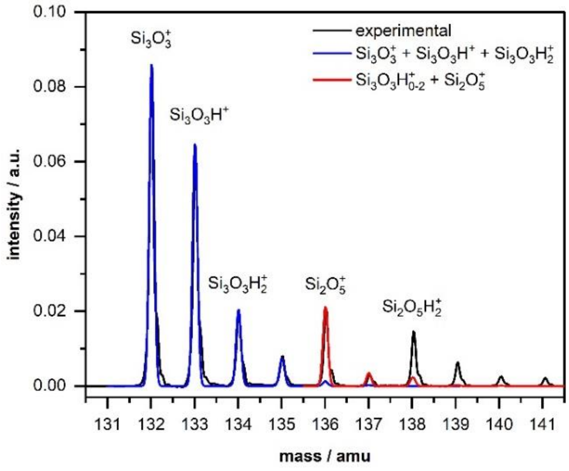

Figure S4. Experimentally obtained mass spectrum (black curves) in the mass range of $Si_2O_5H_2^+$ (m = 138 amu) together with simulated isotope distributions for the bare clusters $Si_3O_3^+/Si_2O_5^+$ as well as the water products $Si_3O_3H^+/Si_3O_3H_2^+/Si_3O_3H_3^+$ and $Si_2O_5H^+/Si_2O_5H_2^+$.

peak corresponding to $Si_2O_3H_2^+$. In the IR-MPD spectrum, recorded on the mass of 106 amu, the lowest intensity band (band I in Figure S3) corresponds to a depletion of $I/I_0 \approx 0.6$, i.e. about 40% of all cluster ions are fragmenting upon irradiation with IR light. Since only about 12% of the cluster ions do not correspond to $Si_2O_3H_2^+$, we conclude that all bands observed in the IR-MPD spectrum arise from $Si_2O_3H_2^+$. However, other species might contribute to the intensity of the peaks, i.e., bands might appear more or less intense in the IR-MPD spectrum.

The situation is even more complex in the mass region of $Si_2O_5^+$ as shown in Figure S4. Upon reaction of water the mass peak at 138 amu grows while the mass peak at 137 amu is not affected. This indicates the formation of $Si_2O_5H_2^+$ (m = 138 amu), whereas $Si_2O_5H^+$ (m = 137 amu) is not formed in noticeable amount. However, between 132 and 135 amu several water products arise (corresponding to $Si_3O_3H_y^+$) whose isotope distributions might also affect the intensity of the mass peak at 138 amu. Therefore, we have considered all these products in our simulation. Based on these simulations we conclude that the mass peak at 138 amu and which is assigned to $Si_2O_5H_2^+$ has contributions from other clusters and water products of about 16% (orange curve at m = 138 amu). Since the lowest intensity peak in the IR-MPD spectrum recorded on this mass (cf. band I in Figure S3) corresponds to a depletion $I/I_0 = 0.75$ (i.e., 25% of all clusters fragment) we conclude that all bands shown in the IR-MPD spectrum arise from $Si_2O_5H_2^+$.

The other masses considered in this manuscript have been evaluated in the same way:

- $Si_3O_6H_2^+$ (m = 182 amu, Figure S5) has a contribution of about 10% from other clusters and water products. For comparison, the lowest intensity band (cf. band I in Figure S3) corresponds to a depletion of $I/I_0 = 0.4$ (i.e., 60% of all clusters fragment). Thus, all bands shown in the IR-MPD spectrum arise from $Si_3O_6H_2^+$.
- $Si_4O_7H_2^+$ (m = 226 amu, Figure S6) has a contribution of about 31% from $Si_4O_7^+$. The lowest intensity bands (cf. bands III and IV in Figure S3) have a depletion of $I/I_0 = 0.2$ (i.e., 80% of all clusters fragment). Thus, all bands shown in the IR-MPD spectrum arise from $Si_4O_7H_2^+$.
- $Si_4O_9H_2^+$ (m = 258 amu, Figure S7) has a contribution of about 25% from $Si_4O_9^+$. The lowest intensity band (cf. band II in Figure S3) has a depletion of $I/I_0 = 0.2$ (i.e., 80% of all clusters fragment). Thus, all bands shown in the IR-MPD spectrum arise from $Si_4O_7H_2^+$.

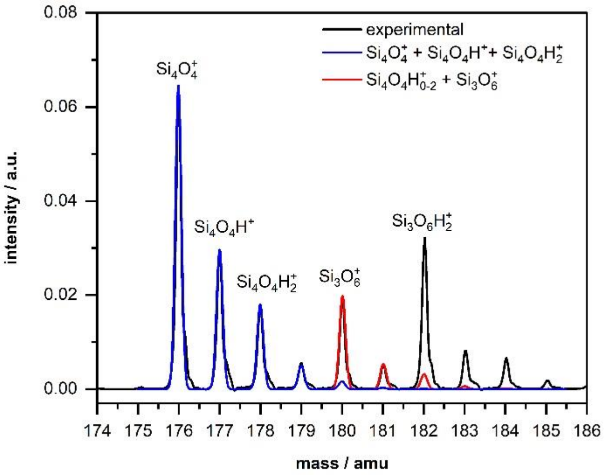

Figure S5. Experimentally obtained mass spectrum (black curves) in the mass range of $Si_3O_6H_2^+$ (m = 182 amu) together with simulated isotope distributions for the bare clusters $Si_4O_4^+/Si_3O_6^+$ as well as the water product $Si_3O_6H^+$.

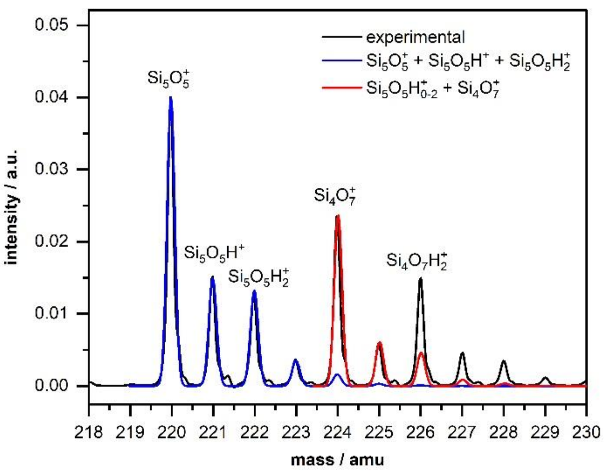

Figure S6. Experimentally obtained mass spectrum (black curves) in the mass range of $Si_4O_7H_2^+$ (m = 226 amu) together with simulated isotope distributions for the bare clusters $Si_5O_5^+$/$Si_4O_7^+$. The water product $Si_4O_7H^+$ does not exist.

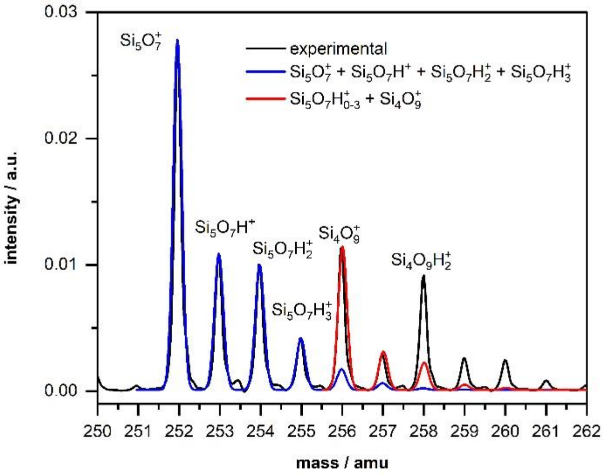

Figure S7. Experimentally obtained mass spectrum (black curves) in the mass range of $Si_4O_9H_2^+$ (m = 258 amu) together with simulated isotope distributions for the bare clusters $Si_5O_7^+$/$Si_4O_9^+$. The water product $Si_4O_9H^+$ does not exist.

S2 - Isomeric structures of $Si_xH_yH_2^+$ clusters

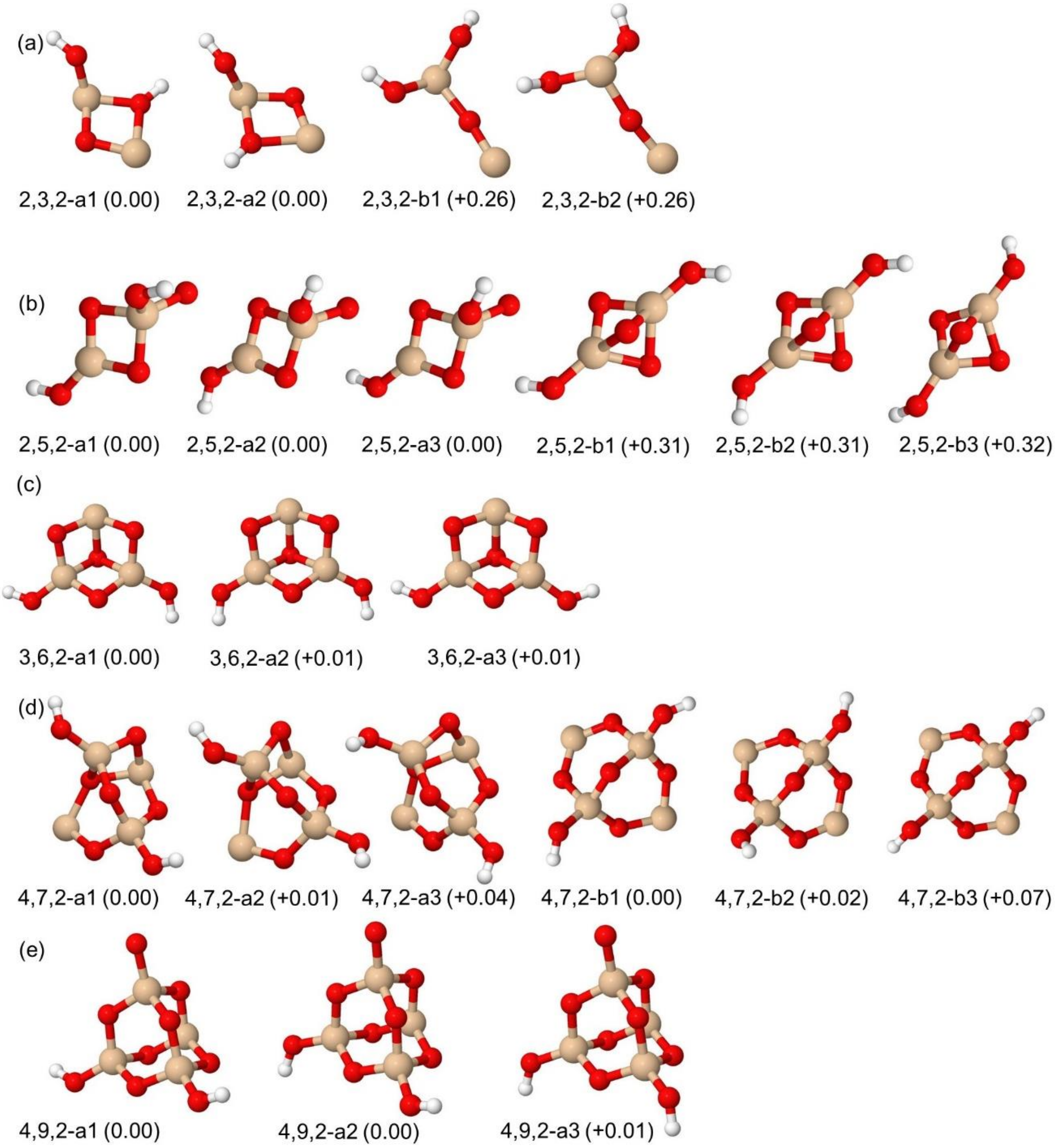

Figure S8. Calculated isomeric structures for $Si_xO_yH_2^+$ complexes (labeled as x,y,2-a# and x,y,2-b#, # = 1-3). Isomers of one group (x,y,2-a# and x,y,2-b#, respectively) have the same cluster core and differ only in the orientation of the hydroxyl groups. Energies (in parentheses) are given in eV. Si, O, and H atoms are depicted as brown, red, and small white spheres, respectively.

S3 - Additional vibrational spectra

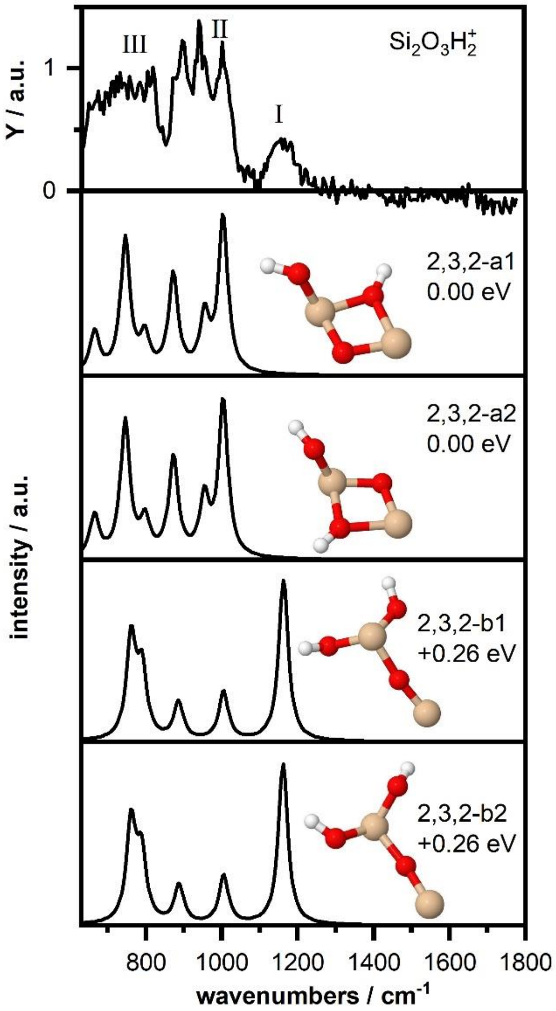

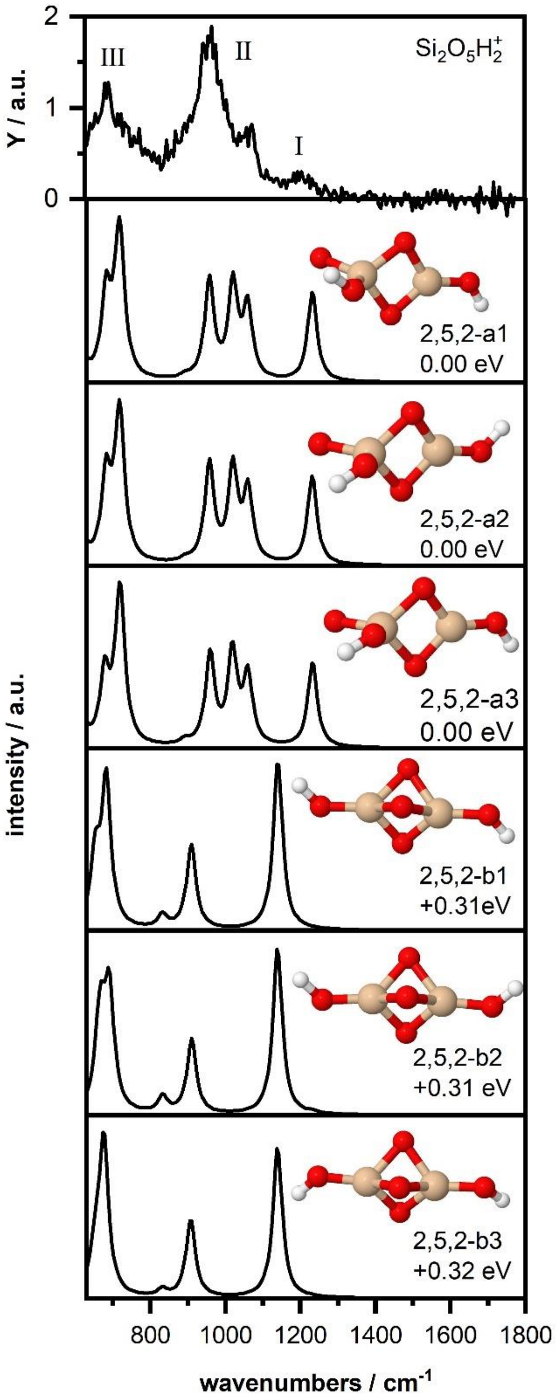

Figure S9. IR-MPD spectrum (top panel) and calculated spectra of different isomers of $Si_2O_3H_2^+$. The IR-MPD spectrum is discussed in detail in the main text. The calculated spectra of the isomers only differing by the orientation of the hydroxyl groups (2,3,2-a1/a2/a3 and 2,3,2-b1/b2, respectively) are very similar. Si, O, and H atoms are depicted as brown, red, and small white spheres, respectively.

Figure S10. IR-MPD spectrum (top panel) and calculated spectra of different isomers of $Si_2O_5H_2^+$. The IR-MPD spectrum is discussed in detail in the main text. The calculated spectra of the isomers only differing by the orientation of the hydroxyl groups (2,5,2-a1/a2/a3 and 2,5,2-b1/b2/b3, respectively) are very similar. Si, O, and H atoms are depicted as brown, red, and small white spheres, respectively.

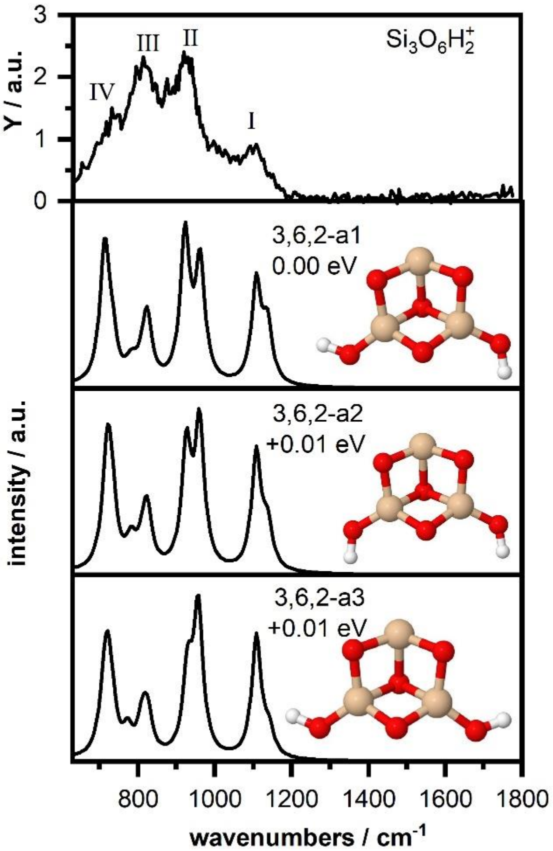

Figure S11. IR-MPD spectrum (top panel) and calculated spectra of different isomers of $Si_3O_6H_2^+$. The IR-MPD spectrum is discussed in detail in the main text. The calculated spectra of the isomers only differing by the orientation of the hydroxyl groups (3,6,2-a1/a2/a3) are very similar. Si, O, and H atoms are depicted as brown, red, and small white spheres, respectively.

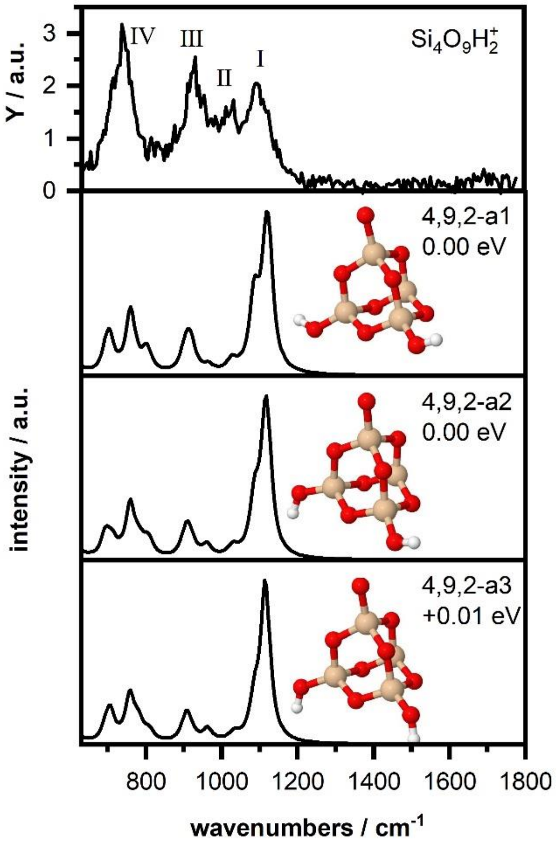

Figure S12. IR-MPD spectrum (top panel) and calculated spectra of different isomers of $Si_4O_9H_2^+$. The IR-MPD spectrum is discussed in detail in the main text. The calculated spectra of the isomers only differing by the orientation of the hydroxyl groups (4,9,2-a1/a2/a3) are very similar. Si, O, and H atoms are depicted as brown, red, and small white spheres, respectively.

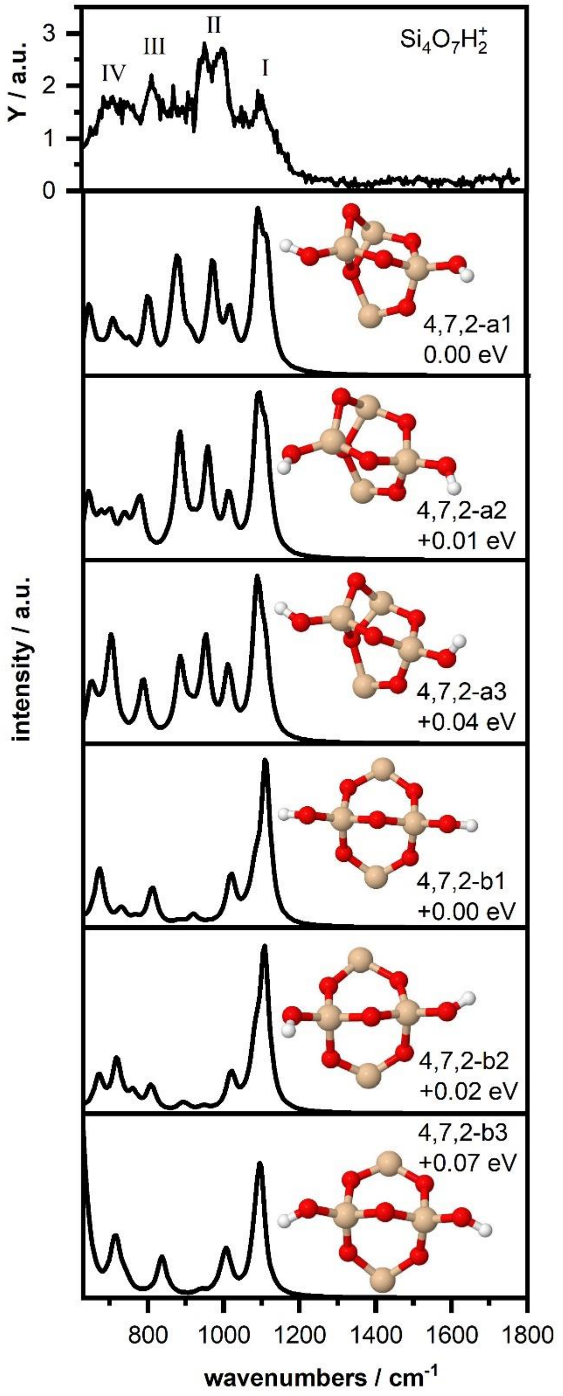

Figure S13. IR-MPD spectrum (top panel) and calculated spectra of different isomers of $Si_4O_7H_2^+$. The IR-MPD spectrum is discussed in detail in the main text. The calculated spectra of the isomers only differing by the orientation of the hydroxyl groups (4,7,2-a1/a2/a3 and 4,7,2-b1/b2/b3, respectively) are very similar. Si, O, and H atoms are depicted as brown, red, and small white spheres, respectively.

S4 - Cosine similarity scores (CSS)

The cosine similarity score are calculated as described in ref. 2. The experimental and theoretical spectra are both treated as vectors in which the frequencies correspond to the vector's dimension and the intensities to each dimension's value. The dot product of these normalized vectors is the cosine similarity score, which is a value between -1 and 1. In this work, we also slightly adapt the calculated spectra following an approach by Kempkes et al.[3] with the intent to make the scores less sensitive to peak intensities and more sensitive to peak positions.

Cluster isomer	Cosine similarity score
**2, 3, 2-a**	**0.89**
2, 3, 2-b	0.66
2, 3, 2-c	0.69
(0.75) 2, 3, 2-a + (0.25) 2, 3, 2-b	0.91
**2, 5, 2-a**	**0.81**
2, 5, 2-b	0.58
(0.75) 2, 5, 2-a + (0.25) 2, 5, 2-b	0.83
**3, 6, 2-a**	**0.88**
3, 6, 2-b	0.80
3, 6, 2-c	0.68
**4, 7, 2-a**	**0.90**
4, 7, 2-b	0.73
4, 7, 2-c	0.81
(0.25) 4, 7, 2-a + (0.75) 4, 7, 2-b	0.91
**4, 9, 2-a**	**0.81**
4, 9, 2-b	0.57
4, 9, 2-c	0.73

S5 - Calculated free energy phase diagrams

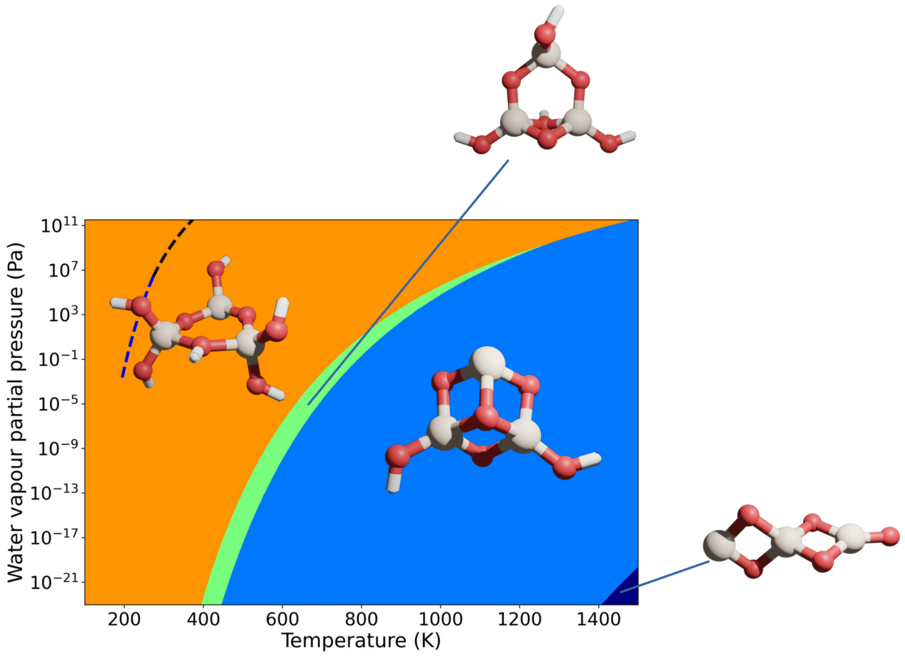

Figure S14. Calculated water vapour partial pressure versus temperature free energy phase diagram for $Si_3O_5(H_2O)_x^+$ (x = 0 - 3) formation. Inset structures indicate the lowest free energy isomer for each coloured region, where the degree of hydration increases from left to right. The dashed curve indicates the water condensation line.

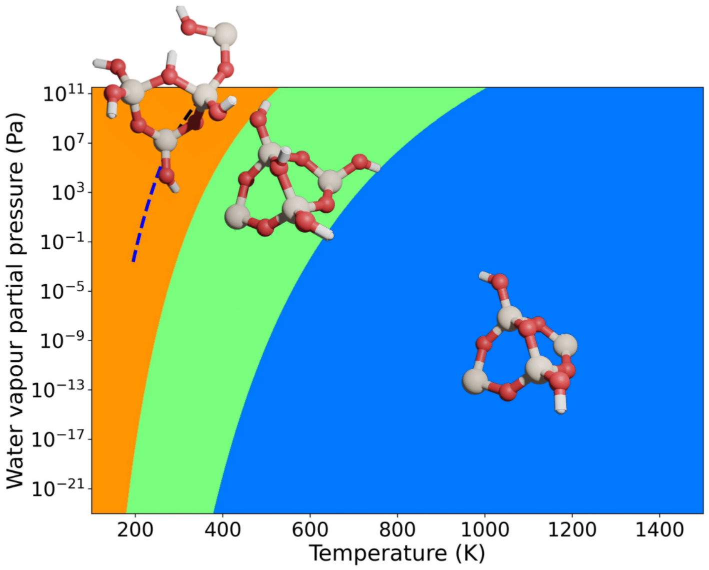

Figure S15. Calculated water vapour partial pressure versus temperature free energy phase diagram for $Si_4O_6(H_2O)_x^+$ (x = 1 - 3) formation. Inset structures indicate the lowest free energy isomer for each coloured region, where the degree of hydration increases from left to right. The dashed curve indicates the water condensation line.

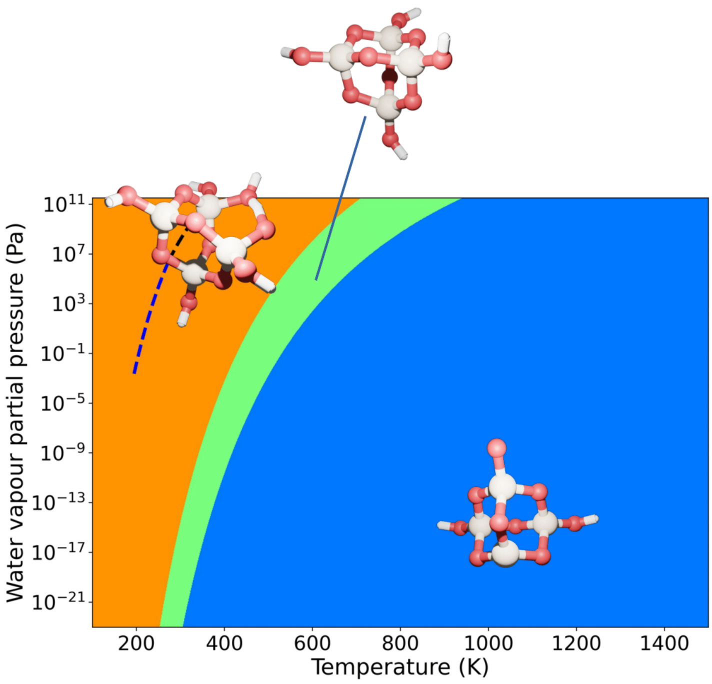

Figure S16. Calculated water vapour partial pressure versus temperature free energy phase diagram for $Si_4O_8(H_2O)_x^+$ (x = 1 - 3) formation. Inset structures indicate the lowest free energy isomer for each coloured region, where the degree of hydration increases from left to right. The dashed curve indicates the water condensation line.

S6 – Cartesian coordinates of lowest energy cluster isomers

DFT-optimised coordinates of the lowest energy isomers are listed below using the .xyz format.

```
7
2, 3, 2-a
Si   -6.24999247    3.77975966    0.11049846
O    -5.07537442    4.03087536   -1.05461168
Si   -4.81287936    5.65897854   -1.05033973
O    -5.71589342    3.19295766    1.50712685
O    -6.17274618    5.54212120    0.27768946
H    -6.00448742    2.35595270    1.88604456
H    -6.56860673    6.14058487    0.92834211

9
2, 5, 2-a
Si   -6.83156532    4.66503967    0.51131740
O    -5.92187516    3.60368968   -0.51014041
Si   -4.83907548    4.77014681   -0.70109970
O    -6.88588280    4.35163617    2.06505316
O    -3.52399500    4.73820486   -1.54116704
H    -7.59384891    3.86395282    2.49944120
H    -2.87257379    5.44426901   -1.65320970
O    -5.59494408    5.84354522    0.21657965
O    -8.29084947    4.95524577   -0.17395456

11
3, 6, 2-a
Si   -4.69181605   15.26172976    5.08259568
Si   -3.50474046   13.57809664    6.44027199
Si   -5.91664864   13.96851723    6.76915959
O    -4.94122778   13.50084288    5.33132614
O    -5.90452346   15.51159770    6.17398290
O    -4.59268627   13.62537728    7.69894757
O    -4.86568590   15.97051000    3.68436836
O    -3.20238970   15.08455247    5.78952089
O    -7.25430981   13.24309417    7.18192985
H    -4.15403262   16.14030646    3.05856483
H    -8.13622932   13.52937541    6.92188222

13
4, 7, 2-a
O    -7.98214333   13.45125140   14.68635234
Si   -8.65869688   12.27313293   13.38195983
Si   -6.56213327   13.10514710   15.70454086
Si   -7.08404546   15.00678874   14.32102649
O    -6.19005218   14.48863650   13.05724137
O    -5.58356041   12.40294012   14.61027331
O    -7.22966266   12.11244176   12.60232844
Si   -5.81024282   12.86730665   13.02506778
O    -6.28273513   14.73076564   15.74439809
```

O	-6.90742294	12.33367321	17.04363677
O	-4.57874266	12.63055174	12.05151724
H	-4.20111840	11.77254300	11.84583042
H	-6.39466386	11.60129121	17.39806705

15
4, 9, 2-a

Si	-22.34711001	3.07987211	9.70643087
O	-19.24391836	2.01208783	9.74546470
Si	-20.01000850	2.80287377	10.89043419
O	-19.33920597	1.87627510	6.98366325
Si	-19.89995368	2.61428277	8.26475733
O	-21.51083672	2.39591798	8.47386739
O	-21.54432168	2.44957432	11.10057551
Si	-20.31306745	5.08428877	9.63878725
O	-19.63675133	4.24065804	8.40905406
O	-21.89423992	4.67024871	9.73916252
O	-20.04430111	6.70182226	9.57067883
O	-23.89128206	2.74196837	9.66533863
H	-18.46402628	2.00481898	6.60945313
H	-24.56496635	3.16467675	10.20379843
O	-19.63338959	4.34327525	11.03231988

References

[1] I. L. Barnes; L. J. Moore; L. A. Machlan; T. J. Murphy; W. R. Shields. *J. Res. Natl. Bur. Stand A Phys. Chem.* 1975, **79a**, 727.

[2] W. Fu, W. S. Hopkins, Applying Machine Learning to Vibrational Spectroscopy. The Journal of Physical Chemistry A, 2017, 122, 167–171.

[3] L. J. M. Kempkes, J. Martens, G. Berden, K. J. Houthuijs, J. Oomens, Investigation of the Position of the Radical in z3-Ions Resulting from Electron Transfer Dissociation Using Infrared Ion Spectroscopy. Faraday Discussions, 2019, 217, 434–452.
